# Type 2 poliovirus detection after global withdrawal of trivalent oral vaccine

**DOI:** 10.1056/NEJMoa1716677

**Published:** 2018-08-30

**Authors:** Isobel M. Blake, Margarita Pons-Salort, Natalie A. Molodecky, Ousmane M. Diop, Paul Chenoweth, Ananda S. Bandyopadhyay, Michel Zaffran, Roland W. Sutter, Nicholas C. Grassly

**Affiliations:** Department of Infectious Disease Epidemiology, Imperial College London, London, UK; Department of Infectious Disease Epidemiology, Imperial College London, London, UK; Department of Infectious Disease Epidemiology, Imperial College London, London, UK; Department of Infectious Disease Epidemiology, Imperial College London, London, UK; Polio Eradication Department, World Health Organization, Geneva, Switzerland; Bill & Melinda Gates Foundation, Seattle, Washington, United States of America; Polio Eradication Department, World Health Organization, Geneva, Switzerland; Polio Eradication Department, World Health Organization, Geneva, Switzerland; Department of Infectious Disease Epidemiology, Imperial College London, London, UK

## Abstract

**Background:**

Mass campaigns with oral poliovirus vaccine (OPV) have brought the world close to wild-type poliovirus eradication. However, to complete eradication, OPV must itself be withdrawn to prevent vaccine-derived poliovirus outbreaks. Synchronized global withdrawal of OPV began with serotype-2 (OPV2) in April 2016, presenting the first test of the feasibility of eradicating all polioviruses.

**Methods:**

We analysed global surveillance data reporting serotype-2 vaccine poliovirus (Sabin-2) and vaccine- derived poliovirus (VDPV2) detection in stool collected during 1 January 2013 through 08 August 2017 from 431,429 children with acute flaccid paralysis in 112 countries and 5485 environmental samples from 4 high-risk countries. We used Bayesian spatiotemporal smoothing and logistic regression to identify and map risk-factors for persistent Sabin-2 and VDPV2 detection.

**Results:**

Detection of Sabin-2 declined rapidly from 3.9% in stool [95% Confidence Interval: 3.5%-4.3%] and 71% [61%-80%] in sewage at the time of OPV2 withdrawal to 0.16% [0.09%-2.7%] and 13% [8%- 20%] at 2 months. However, at 12 months Sabin-2 continued to be detected (0.05% [0.01%-0.13%] in stool, 8% [5%-13%] in sewage) due to OPV2 use in response to VDPV2 outbreaks. Five outbreaks were reported after OPV2 withdrawal, associated with low routine immunisation coverage and population immunity (Odds Ratios 2.59 [1.26-6.33] and 4.65 [1.71-15.28] per 10% absolute decrease).

**Discussion:**

High population immunity has facilitated rapid decline of Sabin-2 after OPV2 withdrawal and restricted circulation of VDPV2 to known areas at high risk of transmission. It will be critical to control these remaining VDPV2 outbreaks before the growth of significant cohorts of susceptible children.

## Introduction

The Global Polio Eradication Initiative (GPEI) has relied on oral poliovirus vaccine (OPV) to bring polio to the brink of eradication. Just 22 cases of poliomyelitis caused by wild poliovirus (WPV) were reported in 2017 (all serotype 1) as of 9 March 2018. OPV is currently used in routine immunisation and mass campaigns among children <5 years old in over 150 countries globally to ensure high levels of population immunity. It is a live-attenuated vaccine (containing Sabin poliovirus strains) that is cheap, easy to administer and, unlike the parenteral inactivated poliovirus vaccine (IPV), replicates in the intestine to induce mucosal immunity that limits further infection and transmission. However, it is genetically unstable and can evolve during replication in the human intestine to regain the neurovirulence and replication characteristics of its parental wild-type strains^1-3^. In rare instances, it can cause vaccine-associated paralytic poliomyelitis ( ~1-2 per million vaccinated) or seed outbreaks of circulating vaccine-derived polioviruses (cVDPV) that cause poliomyelitis (~1 outbreak per 500 million vaccinated)^4^. 

The last naturally occurring case of poliomyelitis caused by serotype 2 WPV was reported in 1999 in India.^5,6^ However, most (>90%) cVDPV poliomyelitis cases reported over the last decade have been caused by serotype 2 (cVDPV2), due in part to declining immunity following widespread use of serotypes 1 and 3 bivalent OPV (bOPV) in supplemental immunization activities (i.e. mass campaigns)^7^. WHO therefore recommended globally synchronised withdrawal of serotype-2 OPV (OPV2) during a two-week period in April 2016 to prevent further cVDPV2 emergences (replacing trivalent with bivalent OPV)^8,9^. To mitigate the risks associated with OPV2 withdrawal, WHO recommended at least one dose of (trivalent) IPV should be used in routine immunisation in all countries. 

A major risk associated with OPV2 withdrawal is occurrence of further cVDPV2 outbreaks, resulting from continued circulation of cVDPV2 seeded by trivalent OPV (tOPV) used prior to withdrawal or accidental use of tOPV after withdrawal. The risk of an outbreak occurring is likely to be highest during the first 12 months following OPV2 withdrawal^10^ . However, if cVDPV2 circulates later in time, the scale of an outbreak will be greater due to the accumulation of children un-immunised against serotype 2. Furthermore, any outbreak of cVDPV2 must be responded to with monovalent OPV2 (mOPV2) because of the superior mucosal immunity induced by this vaccine compared with IPV^11^. Use of mOPV2 could seed more cVDPV2, particularly as time increases since OPV2 withdrawal, risking escalating OPV2 usage and ‘cessation failure’.^12^

The global withdrawal of OPV2 is therefore seen as a major test of the feasibility of eradication of all polioviruses as envisaged by the GPEI^13^. Serotype 2 vaccine poliovirus (i.e. Sabin-2 virus) detection has declined after OPV2 withdrawal^14^, although analysis of data in 17 countries found some unexpected detections during the first 8 months^15^. Here, we analyse the geographic distribution of Sabin-2 and cVDPV2 detected in stool and sewage samples collected from 112 countries over the first 15 months after OPV2 withdrawal. 

## Methods

### Data

Children aged 0-14 years with acute flaccid paralysis (AFP) are reported through a network of healthcare providers and data from clinical and epidemiological investigations recorded in the Polio Information System maintained by the GPEI.^16^ Two stool samples from each AFP case are analysed for the presence of wild, vaccine (Sabin) or vaccine-derived polioviruses using standard protocols^17,18^. Most (99%) AFP is not caused by poliovirus (non-polio AFP) and detection of Sabin poliovirus is usually a coincidental finding rather than an indicator of vaccine-associated paralytic poliomyelitis 19. We analysed epidemiological and laboratory data from AFP cases reported from 112 countries in the African, Eastern Mediterranean, Southeast Asian and European regions with stool samples collected between 1-Jan-2013 and 8-Aug-2017. VDPV2 are defined as vaccine-derived polioviruses that are at least 0.6% divergent from Sabin-2 in the VP1 region, and genetically linked isolates consistent with circulation are classified as cVDPV2^20^.

Environmental surveillance (ES), the systematic collection and testing of sewage samples for polioviruses, is performed in >30 countries to supplement AFP surveillance.^21,22^ We analysed ES data from four high-risk countries (Afghanistan, Pakistan, Nigeria and Kenya) collected between 1-Jan- 2013 and 8-Aug-2017. Samples were tested for polioviruses using standard WHO protocols^23^. The number and spatial distribution of collection sites is shown in Fig S1.

### Statistical analysis

*Sabin-2 detection*: We fit logistic regression models to data on the prevalence of Sabin-2 poliovirus isolated from non-polio AFP cases before OPV2 withdrawal. We assumed the odds of Sabin-2 detection declines as an exponential function of time since the last tOPV campaign in that province. It asymptotically approaches a low constant background level, resulting from either routine vaccination with tOPV or migration of recently vaccinated children from other provinces, estimated using a logit- link offset that is independent of time. Countries were grouped by region in the analysis except for the 3 wild poliovirus endemic countries and India, which were analysed at the national (Afghanistan) or sub-national level (India, Pakistan, Nigeria; Table S1). Fixed effects determining the rate of Sabin-2 decline and the background level were estimated for each population. Data were censored at the time of the next campaign or 6 months, whichever was sooner. We used the same approach for ES data, but with a mixed-effects model to account for repeated observations at each ES site and site-specific variation in sensitivity to detect poliovirus. The models were fitted under a Bayesian framework using the integrated nested Laplace approximation through the R-INLA package^24^ and the R programming language^25^. We used the fitted models to predict the prevalence of Sabin-2 detection after OPV2 withdrawal, accounting for the use of mOPV2 in subsequent outbreak response campaigns by assuming decline after mOPV2 occurs at the same rate as after tOPV (Supplementary Material). 

We also tested the correlation between serotype-2 population immunity in children <36 months old and the estimated time for Sabin-2 prevalence to reach background levels (+0.1%) following a tOPV campaign. Population immunity was estimated at subnational levels over 6-month time periods from vaccination histories of non-polio AFP cases (<36 months old) and estimates of OPV efficacy against serotype-2 poliomyelitis using a spatiotemporal random-effects model as previously described.^26^

*cVDPV2 cases*: We performed univariable and multivariable mixed-effects logistic regression to identify risk factors for whether a province reported cVDPV2 cases after OPV2 withdrawal. The most parsimonious yet adequate model was identified using the ‘widely applicable information criteria’ (WAIC) 27. Full details of the statistical methods and data sources are provided in the Supplementary Appendix.

Data analysis was performed by the first author; the initial draft of the manuscript was written by the first and last authors; and all authors provided final approval for publication of the manuscript.

## Results

### Sabin-2 poliovirus decline before OPV2 withdrawal

**Figure 1:**
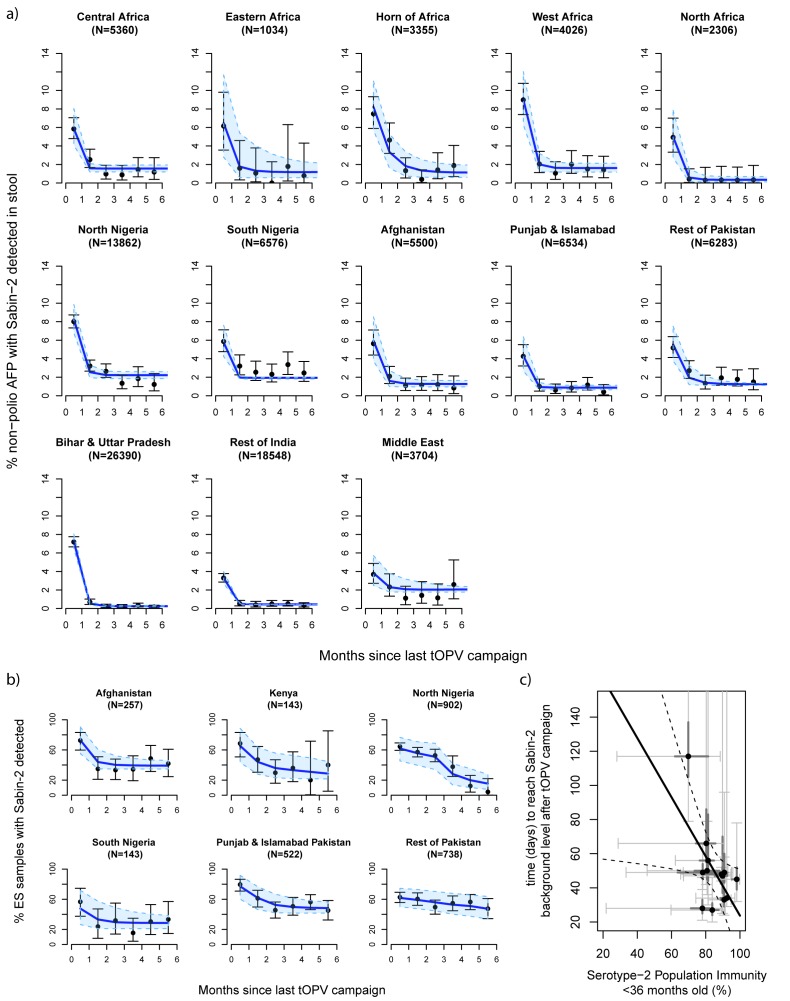


The proportion of non-polio AFP cases positive for Sabin-2 in stool was highest within the first month following a tOPV campaign, and rapidly declined to a low background level (<3%) within 1-2 months of the campaign (Fig 1a). Background levels varied from 0.3%-2.2% and were highest in Nigeria. Decline was slower in the Horn of Africa, North Nigeria, West Africa, Afghanistan and Pakistan (outside of Punjab and Islamabad provinces) such that the odds ratios of detecting Sabin-2 thirty days after the last tOPV campaign (compared to the time of the campaign) were significantly larger in these populations (Table S2). Low serotype-2 immunity appeared to be a risk-factor for persistent detection, with the time for Sabin-2 detection to reach background levels increasing with lower immunity (Figure 1c, r^2^=0.34, p=0.037). Estimated serotype 2 population immunity increased in the majority of countries up until April 2016, from 81.5% on average in July-December 2015 to 88.4% January-April 2016 (Fig S2). 

The proportion of environmental samples positive for Sabin-2 virus also declined after tOPV campaigns, albeit at a significantly slower rate and to a higher background level than in stool samples (Fig 1b, Table S2).

### Sabin-2 poliovirus decline after OPV2 withdrawal

**Figure 2:**
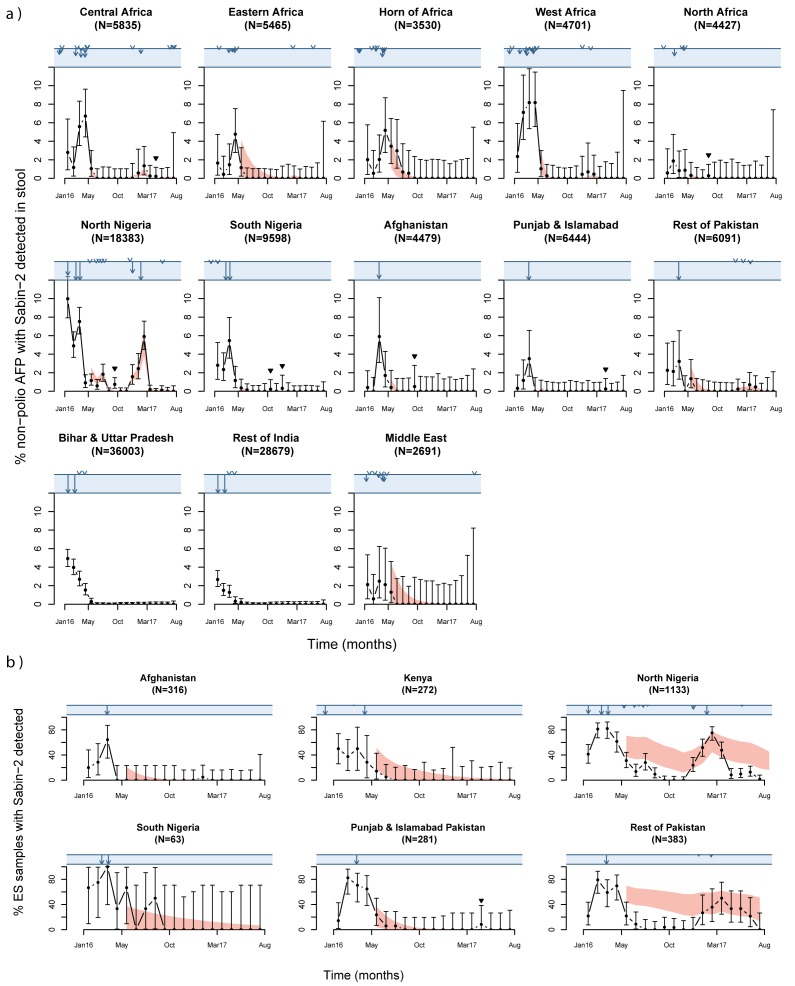


**Figure 3:**
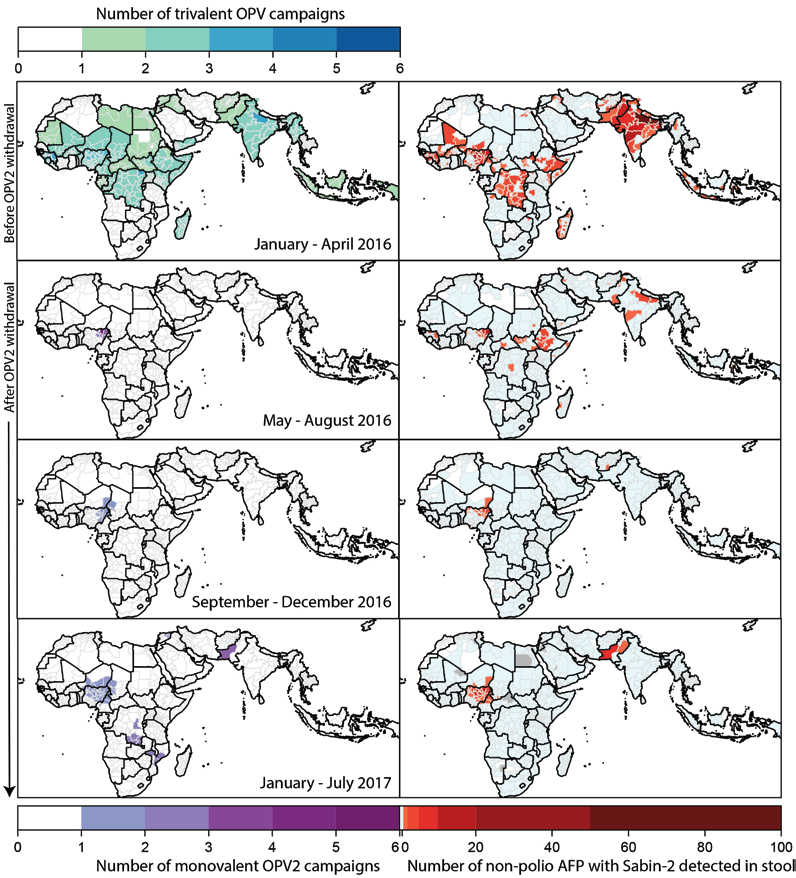


The prevalence of Sabin-2 in stool from children with non-polio AFP rapidly declined in all countries following OPV2 withdrawal in April 2016, from 3.9% in March to 0.16% in June 2016 (chi-squared test, p<0.0001; Fig 2). The geographic distribution became more localised over time and in June 2016 the virus was only detected in children with non-polio AFP in Nigeria, Western Africa and the Horn of Africa (Fig 3, Sabin-2 detections by country are shown in Fig S3-6). These declines and persistence in areas with poorer serotype-2 immunity were consistent with the statistical model of Sabin-2 detection (Fig 2). However, four Sabin-2 detections occurred in Sudan, South Nigeria and Afghanistan between August and December 2016 that were not expected from the statistical model of Sabin-2 detection (Fig 2).

During 2016-17, the number of ES samples collected by month increased over time (Figure S7). The proportions of monthly samples that detected Sabin-2 in all four countries were relatively high (>=20%) prior to OPV2 withdrawal and declined following the last tOPV campaign in each country (Fig 2). The rate of decline was faster than expected from the statistical model of Sabin-2 detection in northern Nigeria and Pakistan (excluding Punjab and Islamabad provinces).

### VDPV2 detection after OPV2 withdrawal

**Figure 4:**
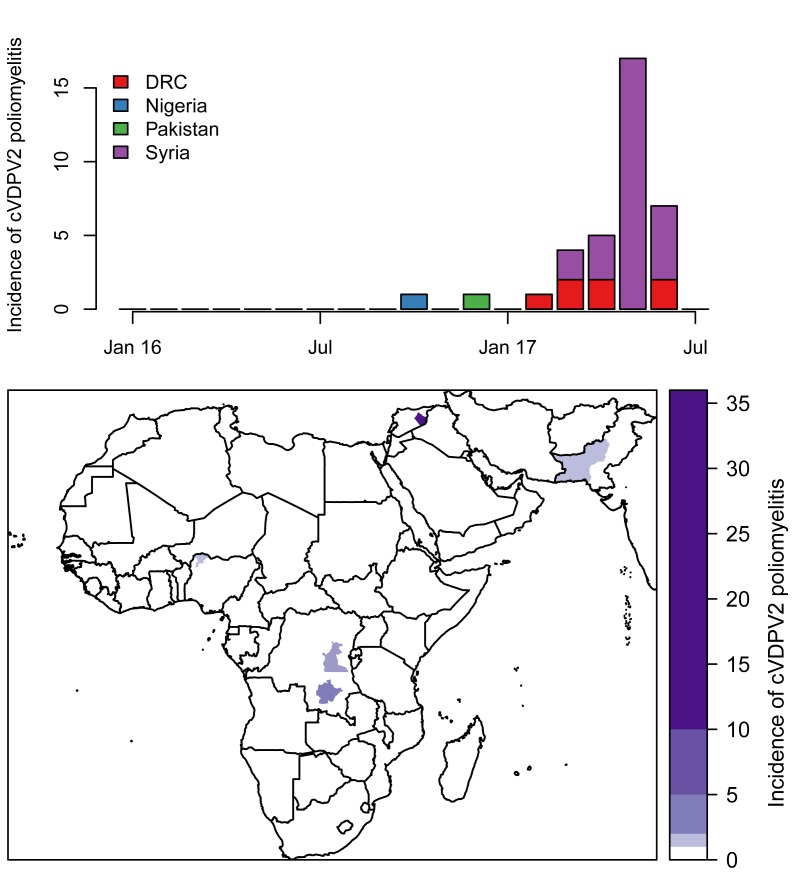


Between April 2016 and 8 August 2017, thirty-six cVDPV2 poliomyelitis cases were reported (Fig 4) linked to five different emergences in four different countries: Nigeria, Pakistan, Democratic Republic of the Congo (DRC) (two separate emergences^28^) and Syrian Arab Republic. Each cVDPV2 outbreak was restricted to a single province, with the majority (27) of cases reported from the Syrian Arab Republic. In univariable analyses, low routine immunisation coverage, serotype-2 population immunity and population density were risk factors for cVDPV2 cases in a province (Table 1). There was no association between cVDPV2 cases and the number or timing of tOPV campaigns prior to OPV2 withdrawal. In the final multivariable model, provinces with poor routine immunisation coverage and low population immunity were more likely to report cVDPV2 cases, such that an absolute decrease of 10% resulted in an increased odds of reporting cVDPV2 cases in a province by a factor of 2.6 and 4.7, respectively (Table 1).

**Table 1:** Univariable and multivariable mixed-effects logistic regression model results for risk factors associated with the reporting of one or more cVDPV2 cases in a province after OPV2 withdrawal (01 May 2016). Data are for all provinces in Nigeria, Pakistan, Syrian Arab Republic and Democratic Republic of the Congo as of 08 August 2017

Fixed Effect	Data source	Univariable Odds Ratio (95% Credible Interval)	Multivariable Odds Ratio (95% Credible Interval)
**Routine immunisation coverage+**	Non-polio AFP cases 12-23 months old* or P3 coverage^29^,‡	1.69 (1.06 – 3.05)	2.59 (1.26 – 6.33)
**Serotype-2 population immunity in the first half of 2016+**	Non-polio AFP cases <36 months old	2.83 (1.28 – 6.80)	4.65 (1.71 – 15.28)
**Number of tOPV campaigns during 6 months prior to OPV2 withdrawal**	Vaccination campaign calendar (Polio Information System)	1.05 (0.44 – 2.18)	-
**Time (days) since the last tOPV campaign prior to April 2016**	Vaccination campaign calendar (Polio Information System)	1.01 (0.81 – 1.27)	-
**Population size (log10)**	Worldpop^30^	0.96 (0.10 – 9.10)	20 (0.49 – 1243)
**Population density (log10 people / km^2^)**	Worldpop^30^ and WHO geodata	0.18 (0.02 – 0.95)	-

+ OR for a 10% absolute decrease

* Pakistan and Syrian Arab Republic

‡ Nigeria and DRC

VDPV2 without evidence of circulation (ambiguous or ‘aVDPV2’) were isolated from 4 poliomyelitis cases and 18 environmental samples after OPV2 withdrawal; 14 of the latter occurred within 4 months of mOPV2 campaigns (Fig S8, Videos 1 and 2).

**Video 1:** 

**Video 2:** 

### Sabin-2 detection after mOPV2 campaigns

mOPV2 campaigns were implemented in several northern states of Nigeria; adjacent areas of Niger, Chad and Cameroon; and Balochistan, Pakistan following detection of cVDPV2 in stool or sewage after OPV2 withdrawal (or shortly before in Borno state, Nigeria^31^); and additionally, in central Mozambique following detection of an aVDPV2 (Fig 3). All these campaigns resulted in subsequent Sabin-2 detection in stool from non-polio AFP cases, as expected from the statistical model of Sabin- 2 detection (Figs 2-3, Videos 1,2). The virus was also detected in a cluster of samples collected from North Nigeria in September 2016 (>1.5 months after a mOPV2 campaign), from southern Chad in April 2017 and from Punjab in Pakistan in June 2017, which were not expected from the statistical model of Sabin-2 detection (Fig 2). The virus was detected for longer following mOPV2 campaigns in ES samples than non-polio AFP stool (Videos 1,2), as predicted by the statistical model of Sabin-2 detection (Table S2). In general, Sabin-2 detections from ES samples in Nigeria occurred within the mOPV2 response areas (Fig 2, Video 2) but in Pakistan, the virus was also detected in ES samples collected from sites outside the response zone and across the border in Kandahar, Afghanistan (Fig 2, Video 1). 

Outbreak response campaigns with mOPV2 occurred in late June and July 2017 in DRC and the Syrian Arab Republic, but as of 8 August 2017 non-polio AFP cases have not yet been reported with completed lab testing of stool from these areas.

## Discussion

The success of global polio eradication depends not only on eradication of wild polioviruses, but of all live polioviruses including the attenuated oral vaccine strains. Although many wealthier countries have successfully switched from OPV to IPV in their routine schedules 32,33, the synchronized withdrawal of OPV2 in April 2016 in all OPV-using countries was a major test of the feasibility of poliovirus eradication. We show here that serotype-2 vaccine poliovirus disappeared rapidly following OPV2 withdrawal, but in a small number of high-risk locations it has persisted – as a result of mOPV2 use in response to VDPV outbreaks or unplanned administration of tOPV from old stocks^14^. We also show that variation in the rate of decline can in part be explained by differences in population immunity, which is likely to affect the duration of individual shedding and the extent of secondary transmission of vaccine poliovirus. This supports the targeted use of preventive campaigns in advance of OPV2 

Outbreaks of cVDPV2 were reported in Nigeria, Pakistan, Syrian Arab Republic and DRC in the period after OPV2 withdrawal. These outbreaks occurred in populations with low routine immunisation coverage and low population immunity against serotype-2 poliomyelitis, in agreement with analyses of VDPV2 emergences and spread in Nigeria.^34^ Whilst highlighting the challenges facing the programme, this clear association with known risk factors for poliovirus transmission and the absence of more widespread cVDPV2 outbreaks, offers support for the GPEI strategy of globally synchronized OPV withdrawal.

GPEI currently recommends at least two high-quality immunisation campaigns with mOPV2 to respond to cVDPV2 outbreaks, given its superior ability to induce mucosal immunity compared with IPV^35^. There is concern that use of mOPV2 threatens eradication of this serotype of poliovirus, given the risk of creating further cVDPV2 in populations with limited routine IPV coverage and growing susceptibility to serotype 2 poliomyelitis^36^. Since OPV2 withdrawal, multiple campaigns with mOPV2 have been implemented in response to cVDPV2 and we show that decline of Sabin-2 after these campaigns has been rapid and in line with predictions from estimates before OPV2 withdrawal.

In terms of geographic spread, in Nigeria there were few detections in stool or the environment outside the response zone after multiple, large-scale mOPV2 campaigns in northern states. In contrast, Sabin-2 was frequently detected outside the response zone in Pakistan, which was initially quite small (600,000 mOPV2 doses across 2,700 km2) compared with Nigeria (2-50 million doses across 32,000- 661,000 km2). This may reflect differences in campaign quality, scale and/or population movement. Isolated aVDPV2s have been detected in sewage samples after mOPV2 campaigns in Nigeria and Pakistan but importantly there is no evidence to date that mOPV2 has led to emergent cVDPV2s with sustained transmission and associated cases of poliomyelitis. Indeed, genetic sequence analysis suggests that all but one cVDPV2 that we report here represent continued transmission of lineages that emerged before OPV2 withdrawal^28^. 

Our study had several limitations. We do not report Sabin-2 isolations from the Americas or the Western Pacific region as these data were not available through the Polio Information System. The reporting rate of AFP varies across populations and our findings are more uncertain in areas with few AFP cases^37^. Environmental surveillance provides important additional data, showing more sustained Sabin-2 detection, consistent with the greater sensitivity of this surveillance method. Expansion of environmental surveillance is an important component of long term polio eradication strategy^22^. Lastly, we do not consider the effect of seasonality on Sabin-2 detection, which may affect the accuracy of our projections because of effects on virus survival and transmission.^19^

In summary, high population immunity at the time of OPV2 withdrawal facilitated rapid disappearance of Sabin-2 and restricted cVDPV2 to areas known to be at high risk of transmission. This is the first test of the GPEI strategy to eradicate all polioviruses including the live vaccine virus. Our findings offer support for the planned withdrawal of bOPV after eradication of wild-type polioviruses is confirmed, provided high immunity and effective surveillance is maintained in high- risk areas. Nonetheless, in 2017, the number of poliomyelitis cases associated with cVDPV2 (96) exceeded those caused by wild poliovirus (22) for the first time and outbreak response campaigns with mOPV2 are continuing in several countries. Timely control of these outbreaks in the context of a growing cohort of children without immunity to type-2 poliovirus is critical to the success of polio eradication. 

## Supplementary Material

Supplementary Appendix

Box Abbreviation: Definition
GPEI: Global Polio Eradication Initiative
WPV: Wild poliovirus
VDPV2: Serotype-2 vaccine-derived polioviruses that are at least 0.6% divergent from Sabin-2 in the VP1 region^20^
cVDPV2: Circulating VDPV2; VDPV2 strain that is genetically linked to another VDPV2 strain indicating person-to-person transmission. A detailed definition is provided in GPEI guidelines^20^. By definition, cVDPV2 refers to an outbreak of VDPV2.
aVDPV2: Ambiguous VDPV2; unlinked VDPV2 isolates that are not from an immunodeficient patient
OPV: Oral polio vaccine
tOPV: Trivalent OPV against serotypes 1, 2, 3
bOPV: Bivalent OPV against serotypes 1, 3
mOPV2: Monovalent OPV against serotype 2
OPV2 withdrawal: Global replacement of tOPV with bOPV within a two-week synchronised period in April 2016 and no further use of OPV2 except for mOPV2 use in outbreak response campaigns
IPV: Inactivated polio vaccine
AFP: Acute flaccid paralysis
ES: Environmental surveillance (systematic testing of sewage for poliovirus)
