# Supplementary material for: Type 2 poliovirus detection after global withdrawal of trivalent oral vaccine
Source: N Engl J Med. Author manuscript; Available in PMC 2018 Aug 30. (PMC5985919; doi:10.1056/NEJMoa1716677)
Supplement: Supplementary Appendix [file NEJMoa1716677_Blake_Supplement.pdf]

Type 2 poliovirus detection after global withdrawal of trivalent oral vaccine

Isobel M Blake[1*], Margarita Pons-Salort[1], Natalie A Molodecky[1], Ousmane M Diop[2], Paul Chenoweth[2], Ananda S Bandyopadhyay[3,] Michel Zaffran[2], Roland W Sutter[2], Nicholas C Grassly[1]

[1]Department of Infectious Disease Epidemiology, St Mary's Campus, Imperial College London, London, UK

[2]Polio Eradication Department, World Health Organization, Geneva, Switzerland

[3]Bill & Melinda Gates Foundation, Seattle, Washington, United States of America

[*]Corresponding author. Email: isobel.blake@imperial.ac.uk

Supplementary Appendix

Table of Contents

Supplementary methods: 3
- Surveillance data 3
- Description of statistical analysis 4

Supplementary Results 9
- Risk Factors for cVDPV2 cases reported after OPV2 withdrawal 9

Supplementary Tables 10
Supplementary Figures 16
References 39

Supplementary methods:

Surveillance data

Children aged 0-14 years with acute flaccid paralysis (AFP) are reported in each country through a network of healthcare providers as part of routine polio surveillance.[1] Information collected from each child includes the dates of onset and notification of paralysis, the date of stool collection, and the province and district of residence. Two stool samples collected 24 hours apart within 14 days of onset of AFP are recommended to be collected from each AFP case and are analysed to determine the presence of wild, vaccine-derived or Sabin polioviruses using Global Polio Laboratory Network protocols[2,3]. The vaccination history of these children and detection of vaccine polioviruses in their stool samples can therefore provide information on vaccine coverage and vaccine poliovirus circulation in the general population[4]. Here we analysed AFP data from countries in the African, Eastern Mediterranean, Southeast Asian and European regions obtained through the Polio Information System maintained by the GPEI. The Polio Information System also records vaccination campaign timing, location and vaccine used (trivalent, bivalent or monovalent OPVs, IPV). Data from AFP cases whose stool samples were collected between 1 January 2013 and 08 August were analysed.

Environmental surveillance (ES), the systematic collection of sewage samples from a given site to test for the presence of polioviruses, is currently performed in over 30 countries to supplement AFP surveillance.[5,6] Here we analyse ES data from four high-risk countries in the Eastern-Mediterranean Region (Afghanistan, Pakistan) and Africa (Kenya, Nigeria) collected between 1 January 2013 and 08 August 2017. The number and spatial distribution of collection sites is shown in Fig S1. Most sites have samples collected monthly. ES samples were collected through the 'grab' method after which samples are concentrated using the two-phase separation method[5,7,8], while additional samples in Pakistan were also collected through the 'Bag Mediated Filtration System'[9]. Samples were then tested for the presence of polioviruses (wild, VDPV and Sabin viruses) using standard WHO protocols[8].

Geographical information system data for boundaries of first and second administrative areas were obtained from the World Health Organization. We refer to first administrative areas as provinces and second administrative areas as districts.

Description of statistical analysis

Statistical model of Sabin-2 detection

i) Detection from non-polio AFP

The probability of Sabin-2 detection from non-polio AFP as a function of time since the last tOPV campaign was estimated through fitting a logistic regression model with a (logit link) offset to the proportion of non-polio AFP cases positive for Sabin-2 in stool, using data from before OPV2 withdrawal and conditioning on cases with stool collected within six months of a campaign. The model was fitted to non-polio AFP data with stool samples collected between 1 January 2013 and 15 April 2016. If $N_{i,t}$ is the total number non-polio AFP in population *i*, *t* days since the last tOPV campaign in the respective province and $X_{i,t}$ is the number of observed non-polio AFP with Sabin-2 detected in stool then $X_{i,t}{\sim}Bin\left(N_{i,t}, p_{i,t}\right)$. The parameter $p_{i,t}$is the probability of detecting Sabin-2 and is defined as $p_{i,t} = q_i + (1 - q_i)\eta_{i,t}$ where $log\left(\eta_{i,t}/\left(1 - \eta_{i,t}\right)\right) = \alpha_i + \beta_i t$. The parameter q_i allows the probability of Sabin-2 detection to asymptotically decline to a non-zero level following a tOPV campaign to account for the fact that routine immunisation with tOPV leads to a low background prevalence of Sabin-2 detection. The background level also accounts for detection of Sabin-2 from migration of individuals recently immunised in other areas. Weak priors were used for the regression parameters ($\alpha{\sim}Norm(0,0),\ \beta{\sim}Norm(0,0.001), q{\sim}Norm(-1,0.0001)$, where the distribution is defined as $Norm(mean, precision)$). The regression model was fitted separately for each population (populations are defined in Table S1) to allow estimating a separate value of q_i per population. A summary of model parameters is given in Table S3. The fit of the model was assessed through plotting the observed data against the posterior predictive distribution, aggregating at the monthly level (Figure 1). In addition, the distribution of the binned residuals (observed – posterior predictive distribution) by month was plotted (Figure S9), as described by [11]. No trends in the binned residuals occurred.

We tested the correlation across populations between the estimated time (days) needed for the prevalence of Sabin-2 to reach background levels (+ 0.1%) following a tOPV campaign and serotype-2 population immunity. Serotype-2 population immunity was estimated at subnational levels over 6-

month time periods from vaccination histories of non-polio AFP cases (<36 months old) and serotype-specific estimates of OPV efficacy against serotype-2 poliomyelitis using a spatiotemporal random-effects model as previously described[10]. Each non-polio AFP case in each population was assigned the immunity estimate for six-month period the stool sample was collected in and the average population immunity was calculated as the median estimate across AFP cases in each population (where populations are defined as in Table S1). To assess general trends in immunity over time across all considered countries the population weighted mean and standard deviation were calculated using population size data from Worldpop[12].

The statistical model of Sabin-2 detection was then used to predict the expected probability ($\sigma_{\tau,t,i}$) of Sabin-2 detection following OPV2 withdrawal: $\sigma_{\tau,t,i} = r_{i,\tau} + (1 - r_{i,\tau})\eta_{i,t}$ where $log(r_{i,\tau}/(1 - r_{i,\tau})) = log(q_i/(1 - q_i)) + \beta_i\tau$, to allow for a decline in Sabin-2 detection from the final children immunised with tOPV through routine immunisation (compared to a constant value above), with τ being the number of days since OPV2 withdrawal (1 May 2016), and *t* the number of days since the last tOPV or mOPV2 campaign. The model assumes that the rate in the decline of Sabin-2 from the final children immunised with tOPV through routine immunisation occurs at the same rate as the decline following tOPV campaigns (hence $\beta_i$is multiplied by τ in the linear predictor of $r_{i,\tau}$). The model also assumes that the change in prevalence of Sabin-2 following an outbreak response mOPV2 campaign after OPV2 withdrawal is similar to that following a tOPV campaign prior to OPV2 withdrawal. A summary of additional model parameters is given in Table S4. Latin hypercube sampling[13] was performed to efficiently sample $\eta_{i,t}$, β_i, and q_i from their posterior marginal distributions as it was not possible to jointly sample these within the R-INLA framework. The predictions were performed from 1000 samples and the 2.5% and 97.5% quantiles of the simulations were compared to the observed proportion of non-polio AFP cases with Sabin-2 detection following the OPV2 withdrawal. Unexpected Sabin-2 detections were defined when the observations were outside the 95% prediction interval.

ii) Detection from ES samples

A similar statistical model was fitted to the ES data whereby $X_{i,t,j}$ represents the number of ES samples with Sabin-2 detected from area i, t days since the last tOPV campaign from site j, and $N_{i,t,j}$ the number of samples tested. The term area refers to either Afghanistan, Kenya, North Nigeria, South Nigeria, Punjab and Islamabad provinces in Pakistan or the remainder of Pakistan. In addition, a per-site random effect was included in this model to account for site-specific variation. Therefore $log(\eta_{i,t,j}/(1-\eta_{i,t,j})) = \alpha_i + \beta t + \mu_j$, where μ_j was assumed to be Normally distributed with mean 0 and estimated variance σ^2. Samples that were scheduled but not collected were not included in the analysis. As described above, the model assumes independence between provinces. The fit of the model was assessed through plotting the observed data against the posterior predictive distribution, aggregating at the monthly level (Figure 1). In addition, the distribution of the residuals (observed – posterior predictive distribution) by month was plotted (Figure S10). When comparing the observed decline of Sabin-2 to the predicted decline, ES sites that began sampling after OPV2 withdrawal were not included (Fig 2).

Risk factors of cVDPV2 cases post OPV2 withdrawal

The emergence and spread of cVDPV2 has been previously found to occur in populations with poor routine immunisation coverage, low population immunity and high birth rates [14]. In this work we explore whether provinces that report at least one cVDPV2 case after OPV2 withdrawal occurred in places that would be considered high risk (the number of cases by province is given in Table S5).

Provinces, $X_{i,j}$, in each country, j, were classified as either reporting or not reporting cVDPV2 cases post OPV2 withdrawal and the log odds of reporting cases was assumed to be a function of previously investigated covariates:[14] [routine immunisation coverage in the given province (r_i), population immunity (m_i), log(10) of population size (q_i), log(10) of population density (f_i), number of tOPV campaigns between October 2016 and March 2017 (b_i), and the time since the last tOPV campaign prior to April 2016 (v_i)], allowing for a random effect by country, such that the full model is written as:

$$X_{i,j} \sim Bern(p_{i,j})$$

$$logit(p_{i,j}) = \alpha + \beta_1 r_i + \beta_2 m_i + \beta_3 q_i + \beta_4 f_i + \beta_5 b_i + \beta_6 v_i + \mu_j$$

$$\mu_j \sim Norm(0, \sigma^2)$$

Routine immunisation coverage was defined as the smoothed proportion of non-polio AFP cases in a given province who reported receiving 3 OPV doses through routine immunisation aged between 12-24 months for Pakistan and Syria (see Molodecky *et al* for a full description of the methods[15]). Estimates of routine immunisation coverage from the first half of 2017 were used as these reflect the actual routine immunisation coverage of the first half of 2016. AFP cases reported from African countries do not report OPV doses received through routine immunisation separately to doses received through immunisation campaigns therefore an alternative source of data for routine immunisation coverage for Nigeria and DRC was required. We used coverage estimates of three doses of Diphtheria-tetanus-pertussis (DTP3) from the most recent Demographic Health Surveys (2013) which have been spatially smoothed to a 5x5km resolution[16]. DTP3 is administered at a similar time to the third dose of OPV in the routine immunisation schedule. The mean coverage at the province level was extracted from the raster data using the R 'velox' package[17].

The method to estimate serotype-2 population immunity against poliomyelitis is referred to above. The province estimates for the first half of 2016 were used in the regression to reflect immunity that would have allowed VDPV2 that emerged pre OPV2 withdrawal to circulate into the post-withdrawal time period. Province estimates for Nigeria and Pakistan were obtained by taking the mean across relevant districts.

Sub-national estimates of the number of annual births were not available for all the considered countries (i.e. Syrian Arab Republic) and therefore we use population size as a proxy measure for this risk factor. For each country the 2015 estimated population size from spatially smoothed surfaces[12,18] were aggregated the province level using the R 'velox' package.[17] Note that the population estimates for Syria are based upon census data collected prior to the recent conflict in this country.

Univariable analyses were performed followed by multivariable model selection using the ‘widely applicable information criteria’ (WAIC)[19]. All analyses were performed using the R-INLA package.[20]

Supplementary Results

Risk Factors for cVDPV2 cases reported after OPV2 withdrawal

A map of the routine immunisation coverage per province is given in Figure S11. Routine immunisation coverage was estimated to be variable both between and within countries. The areas with the lowest routine immunisation coverage were northern Syria, Western Pakistan, central DRC and northern Nigeria. The total population size also was variable across provinces, with the highest population located in eastern Pakistan (Figure S12).

Multivariable model selection was performed to determine the most parsimonious yet adequate model (Table S6). The model which included routine immunisation coverage, population immunity and population size resulted in the lowest WAIC. The estimated variance for the country level random effect in the final model was 0.000 (95% credible interval: 0.000 - 0.002).

Supplementary Tables

Table S1 Definitions of regional groupings used in the analysis of non-polio AFP data

Geographic Area	Included countries / provinces
**Central Africa**	Angola, Chad, Cameroon, Democratic Republic of the Congo, Republic of Congo, Central African Republic, Gabon, Equatorial Guinea, São Tomé and Príncipe
**Eastern Africa**	Mozambique, Malawi, Zimbabwe, Uganda, Zambia, Tanzania, Rwanda, Mauritius, Madagascar, Burundi, Comoros, Reunion, Seychelles
**Horn of Africa**	Ethiopia, Eritrea, South Sudan, Kenya, Djibouti, Somalia
**Western Africa**	Benin, Guinea, Ghana, Cote D'Ivoire, Senegal, Togo, Mali, Burkina Faso, Niger, Sierra Leone, Liberia, Gambia, Guinea Bissau, Cape Verde
**North Africa**	Sudan, Egypt, Tunisia, Morocco, Libya, Algeria, Mauritania
**North Nigeria**	Katsina, Sokoto, Jigawa, Borno, Bauchi, Taraba, Zamfara, Niger, Yobe, Kaduna, Adamawa, Gombe, Benue, Nasarawa, Plateau, FCT Abjua
**South Nigeria**	Remainder of Nigeria
**Middle East**	Iran, Iraq, Jordan, Lebanon, Saudi Arabia, Syria, West Bank and Gaza Strip, Yemen

Table S2 i) Estimated Odds Ratio (OR) of Sabin-2 detection 30 days after a tOPV campaign compared to at the time of a tOPV campaign, and ii) estimates of Sabin-2 background prevalence (%) as a result of routine immunisation with tOPV or migration from other areas recently vaccinated; Left: from non-polio AFP cases with stool samples collected between Jan 2013- Apr 2016; Right: from environmental surveillance samples collected between Jan 2013 – Apr 2016 (CrI indicates the Credible Interval)

	**Stool from non-polio AFP**			**Environmental surveillance samples**		
	**OR (95% CrI)**	**Background prevalence (%) (95% CrI)**	**N**	**OR (95% CrI)**	**Background prevalence (%) (95% CrI)**	**N**
Central Africa	0.00 (0.00, 0.03)	1.5 (1.2, 1.9)	5,360	-	-	-
East Africa+	0.08 (0.00, 0.60)	1.0 (0.6, 1.9)	1,034	0.40 (0.16, 0.72)	29.0 (24.3, 38.2)	143
Horn of Africa	0.30 (0.14, 0.45)	1.1 (0.6, 1.8)	3,355	-	-	-
West Africa	0.04 (0.01, 0.12)	1.6 (1.2, 2.1)	4,026	-	-	-
North Africa	0.03 (0.00, 0.14)	0.3 (0.1, 0.7)	2,306	-	-	-
North Nigeria	0.04 (0.01, 0.09)	2.2 (1.8, 2.6)	13,862	0.71 (0.63, 0.79)	0.0 (0.0, 2.4)	902
South Nigeria	0.00 (0.00, 0.03)	2.1 (1.9, 2.1)	6,576	0.05 (0.00, 0.57)	28.0 (21.2, 36.1)	143
Afghanistan	0.05 (0.01, 0.19)	1.3 (1.0, 1.6)	5,500	0.10 (0.01, 0.41)	40.5 (36.6, 47.0)	257
Punjab & Islamabad, Pakistan	0.01 (0.00, 0.06)	0.9 (0.6, 1.1)	6,534	0.24 (0.08, 0.51)	47.7 (41.3, 53.6)	522
Remainder of Pakistan	0.13 (0.02, 0.41)	1.3 (1.2, 1.4)	6,283	0.88 (0.81, 0.96)	0.0 (0.0, 1.8)	738
Bihar & Uttar Pradesh, India*	0.03 (0.02, 0.04)	0.3 (0.2, 0.3)	26,390	-	-	-
Remainder of India*	0.01 (0.00, 0.03)	0.4 (0.4, 0.5)	18,548	-	-	-
Remainder EMRO	0.09 (0.00, 0.64)	1.9 (1.8, 2.1)	3,704	-	-	-

*Data only available from 2014 onwards, + ES samples are from Kenya only

Table S3 Statistical model parameters of Sabin-2 detection prior to OPV2 withdrawal

Parameter	Definition	Inclusion in model of Sabin-2 detection from non-polio AFP stool	Inclusion in model of Sabin-2 detection from environmental surveillance samples
$\boldsymbol{i}$	Population index	✓	✓
$\boldsymbol{j}$	Environmental site index	NA	
$\boldsymbol{t}$	Number of days since the last tOPV campaign in the respective province	✓	✓
$\boldsymbol{q_i}$	Constant background probability of Sabin-2 detection to account for routine immunisation with tOPV or migration from recently vaccinated other areas	✓	✓
$\boldsymbol{\eta_{i,t}}$	Probability of Sabin-2 detection as a function of time since the last tOPV campaign	✓	✓ (Also indexed by j)
$\boldsymbol{logit(\eta_{i,t})}$	Linear predictor	✓	✓ (Also indexed by j)
$\boldsymbol{\alpha_i}$	Intercept in linear predictor	✓	✓
$\boldsymbol{\beta_i}$	Slope coefficient for the covariate of *t* in the linear predictor	✓	✓
$\boldsymbol{\mu_j}$	Random effect of environmental site *j*	NA	✓
$\boldsymbol{p_{i,t}}$	Overall probability of Sabin-2 detection prior to OPV2 withdrawal $[p_{i,t} = q_i + (1 - q_i)\eta_{i,t}]$	✓	✓ (Also indexed by j)

Table S4 Additional parameters used in predicting from the statistical model of Sabin-2 detection after OPV2 withdrawal

Parameter	Definition	Inclusion in model of Sabin-2 detection from non-polio AFP stool	Inclusion in model of Sabin-2 detection from environmental surveillance samples
$\boldsymbol{\tau}$	Number of days since global OPV2 withdrawal (1 May 2016)	✓	✓
$\boldsymbol{t}$	Number of days since the last OPV2 campaign (either tOPV before 1 May 2016 or mOPV2 after 1 May 2016) in the respective province	✓	✓
$\boldsymbol{r_{i,\tau}}$	Probability of Sabin-2 detection from children immunised with tOPV through routine immunisation that continue excreting Sabin-2 for a limited period after 1 May 2016 that declines as a function of τ	✓	✓
$\boldsymbol{\sigma_{\tau,t,i}}$	Overall probability of Sabin-2 detection after OPV2 withdrawal $[p_{i,t} = \boldsymbol{r}_{i,\tau} + (1 - \boldsymbol{r}_{i,\tau})\eta_{i,t}]$	✓	✓ (Also indexed by j)

Table S5 Reported number of cVDPV2 cases in each province of the four countries with cVDPV2 outbreaks confirmed following OPV2 withdrawal. [Note two different outbreaks occurred in the Democratic Republic of the Congo[21]]. Data are as of as of 08 August 2017

Country	Province	Number of cVDPV2 cases
**NIGERIA**	ABIA	0
	ADAMAWA	0
	AKWA IBOM	0
	ANAMBRA	0
	BAUCHI	0
	BAYELSA	0
	BENUE	0
	BORNO	0
	CROSS RIVER	0
	DELTA	0
	EBONYI	0
	EDO	0
	EKITI	0
	ENUGU	0
	FCT, ABUJA	0
	GOMBE	0
	IMO	0
	JIGAWA	0
	KADUNA	0
	KANO	0
	KATSINA	0
	KEBBI	0
	KOGI	0
	KWARA	0
	LAGOS	0
	NASARAWA	0
	NIGER	0
	OGUN	0
	ONDO	0
	OSUN	0
	OYO	0
	PLATEAU	0
	RIVERS	0
	SOKOTO	1
	TARABA	0
	YOBE	0
	ZAMFARA	0
**SYRIAN ARAB REPUBLIC**	QUNEITERA	0
	ALEPPO	0
	DAMASCUS	0
	DARA	0

	DEIR_AL_ZOUR	27
	EDLEB	0
	HAMA	0
	HASAKEH	0
	HOMS	0
	LATTAKIA	0
	RURAL_DAMASCUS	0
	RAQUA	0
	SWIEDA	0
	TARTOUS	0
**PAKISTAN**	AJK	0
	BALOCHISTAN	1
	GILGIT BALTISTAN	0
	ISLAMABAD	0
	PUNJAB	0
	SINDH	0
	FATA	0
	KHYBER PAKHTOON	0
**DEMOCRATIC REPUBLIC OF THE CONGO**	BAS UELE	0
	EQUATEUR	0
	HAUT KATANGA	0
	HAUT LOMAMI	5
	ITURI	0
	KASAI	0
	KASAI CENTRAL	0
	KASAI ORIENTAL	0
	KINSHASA	0
	KONGO CENTRAL	0
	KWANGO	0
	KWILU	0
	LOMAMI	0
	LUALABA	0
	MAI NDOMBE	0
	MANIEMA	2
	MONGALA	0
	NORD KIVU	0
	NORD UBANGI	0
	ORIENTAL	0
	SANKURU	0
	SUD KIVU	0
	SUD UBANGI	0
	TANGANIKA	0
	TSHOPO	0
	TSHUAPA	0

Table S6 Model comparison based upon the WAIC for different mixed-effects logistic models of cVDPV2 cases at the province level following OPV2 withdrawal

Model	WAIC
$logit(p_{i,j}) = \alpha + \beta_1 r_i + \beta_2 m_i + \beta_3 q_i + \beta_4 f_i + \beta_5 b_i + \beta_6 v_i + \mu_j$	37.3
$logit(p_{i,j}) = \alpha + \beta_1 r_i + \beta_2 m_i + \beta_3 q_i + \beta_4 f_i + \beta_5 b_i + \mu_j$	37.1
$logit(p_{i,j}) = \alpha + \beta_1 r_i + \beta_2 m_i + \beta_3 q_i + \beta_4 f_i + \mu_j$	34.7
$logit(p_{i,j}) = \alpha + \beta_1 r_i + \beta_2 m_i + \beta_3 q_i + \mu_j$	**33.8**
$logit(p_{i,j}) = \alpha + \beta_1 r_i + \beta_2 m_i + \beta_4 f_i + \mu_j$	36.5
$logit(p_{i,j}) = \alpha + \beta_1 r_i + \beta_2 m_i + \mu_j$	34.7
$logit(p_{i,j}) = \alpha + \beta_1 r_i + \beta_3 q_i + \mu_j$	38.9
$logit(p_{i,j}) = \alpha + \beta_2 m_i + \beta_3 q_i + \mu_j$	38.2

Supplementary Figures

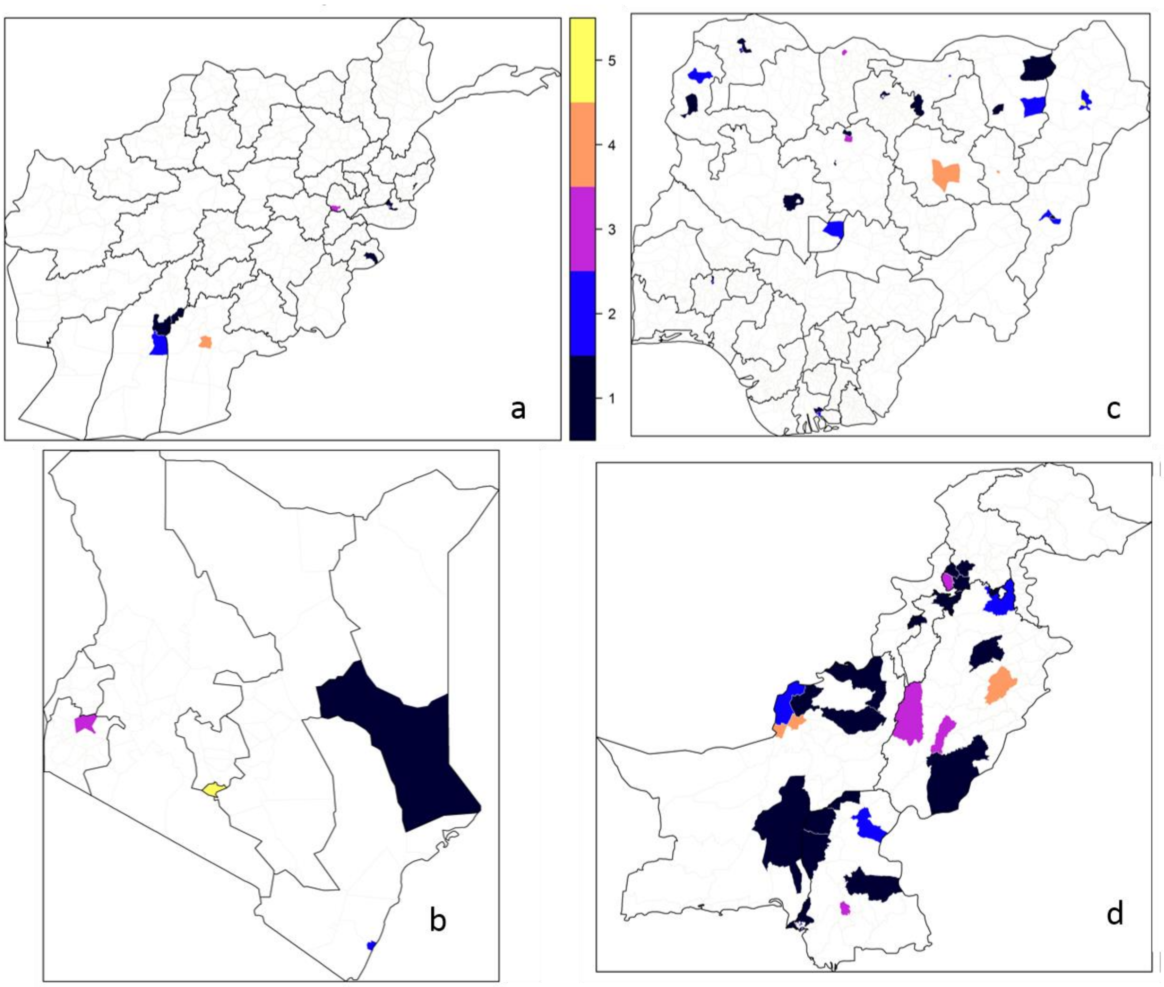

Figure S1 Number of ES sites per district in Afghanistan (a), Kenya (b), Nigeria (c) and Pakistan (d) in 2016. Province divisions are shown in black whilst district divisions are shown in light grey.

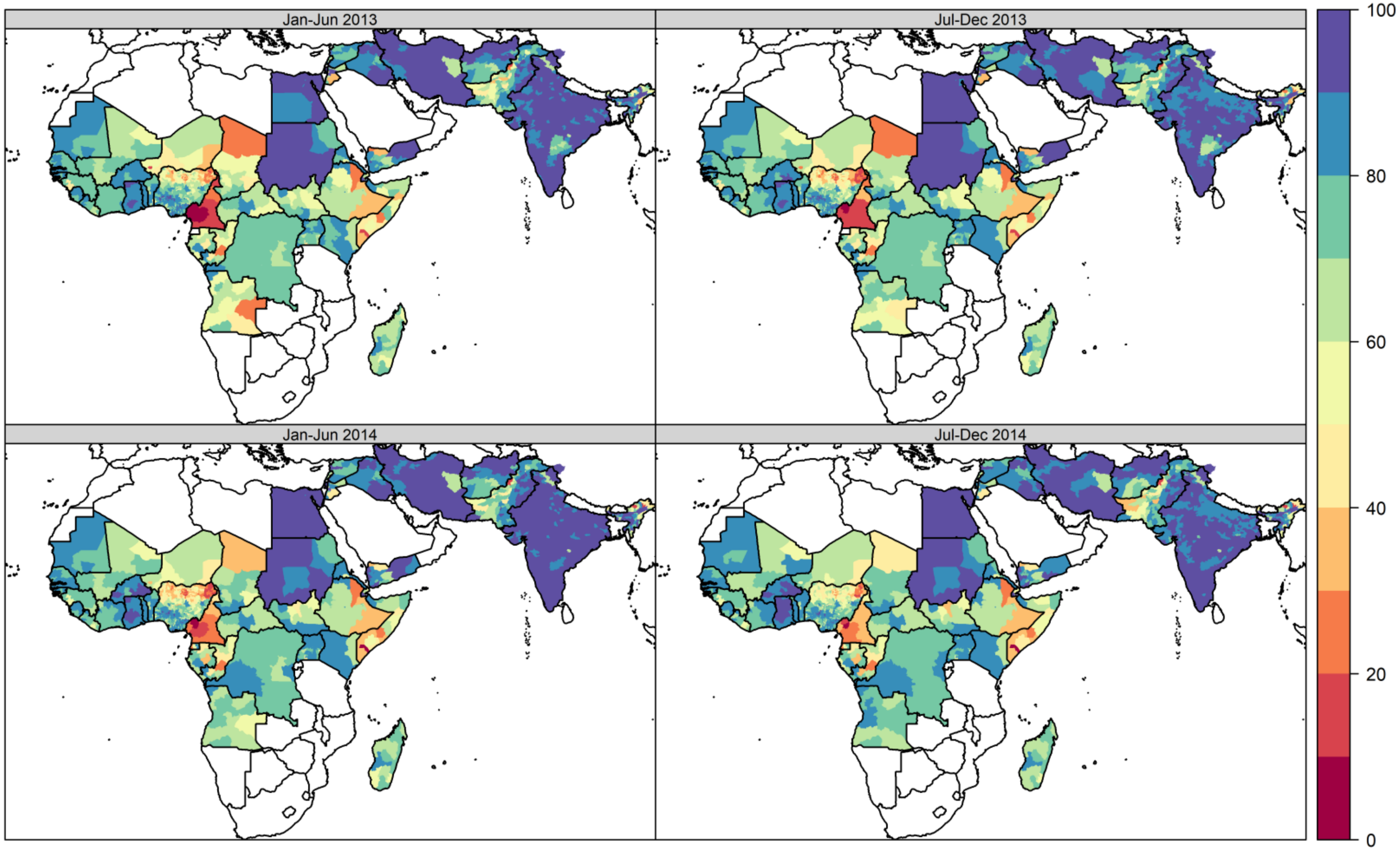
Jan-Jun 2013
Jul-Dec 2013
Jan-Jun 2014
Jul-Dec 2014
100
80
60
40
20
0

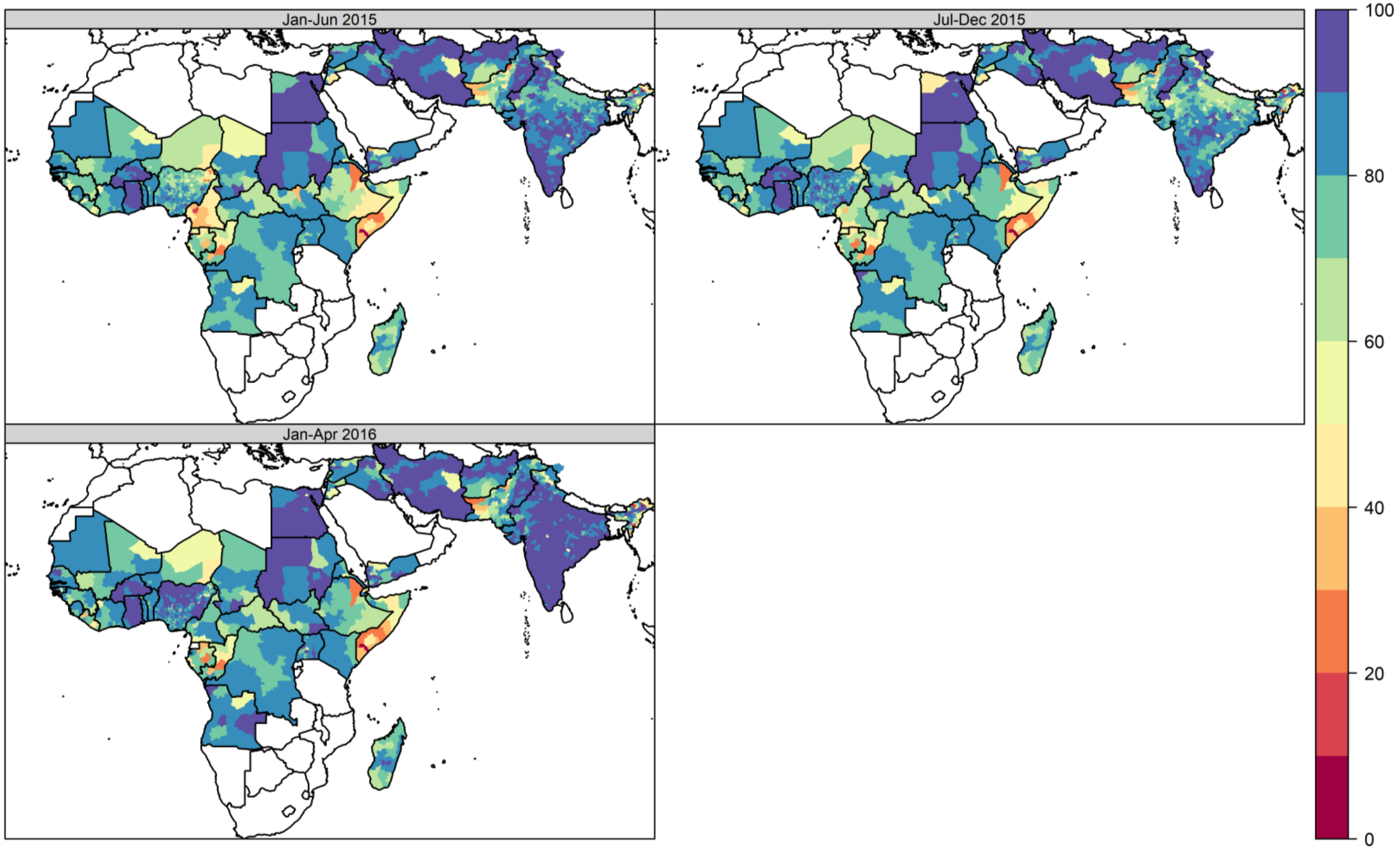

Figure S2 Subnational estimates of population immunity against serotype-2 poliomyelitis (%) in children <36 months old over six-month time periods. Population immunity was estimated for countries that received at least one tOPV campaign between 2013 and 2015 and that were part of a region which detected Sabin-2 at least 2 months after OPV2 withdrawal. Immunity was estimated at the district level in Nigeria, Pakistan and India but at the province level elsewhere (due to relatively low number of non-polio AFP cases).

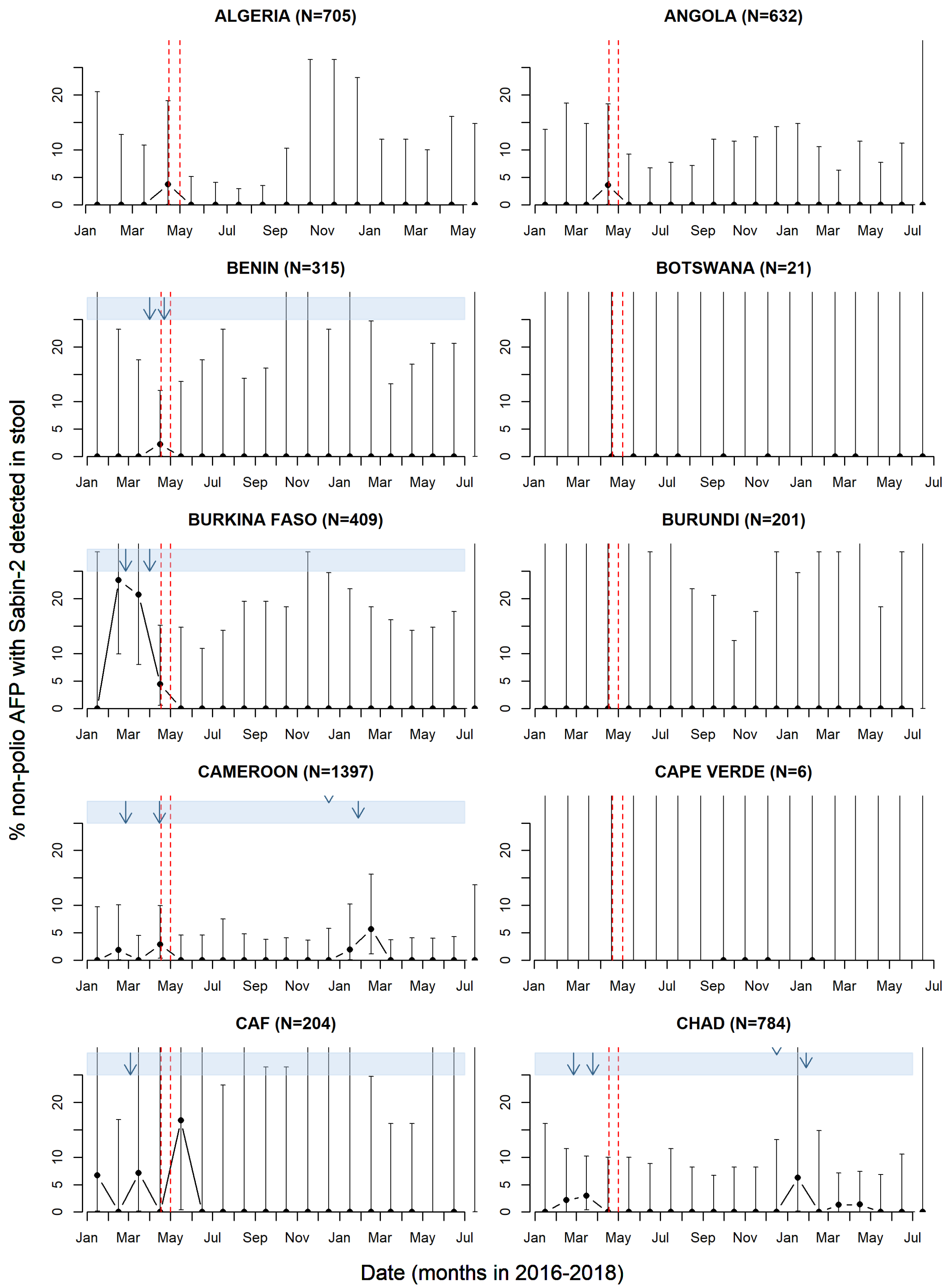
ALGERIA (N=705)
ANGOLA (N=632)
BENIN (N=315)
BOTSWANA (N=21)
BURKINA FASO (N=409)
BURUNDI (N=201)
CAMEROON (N=1397)
CAPE VERDE (N=6)
CAF (N=204)
CHAD (N=784)
% non-polio AFP with Sabin-2 detected in stool
Date (months in 2016-2018)

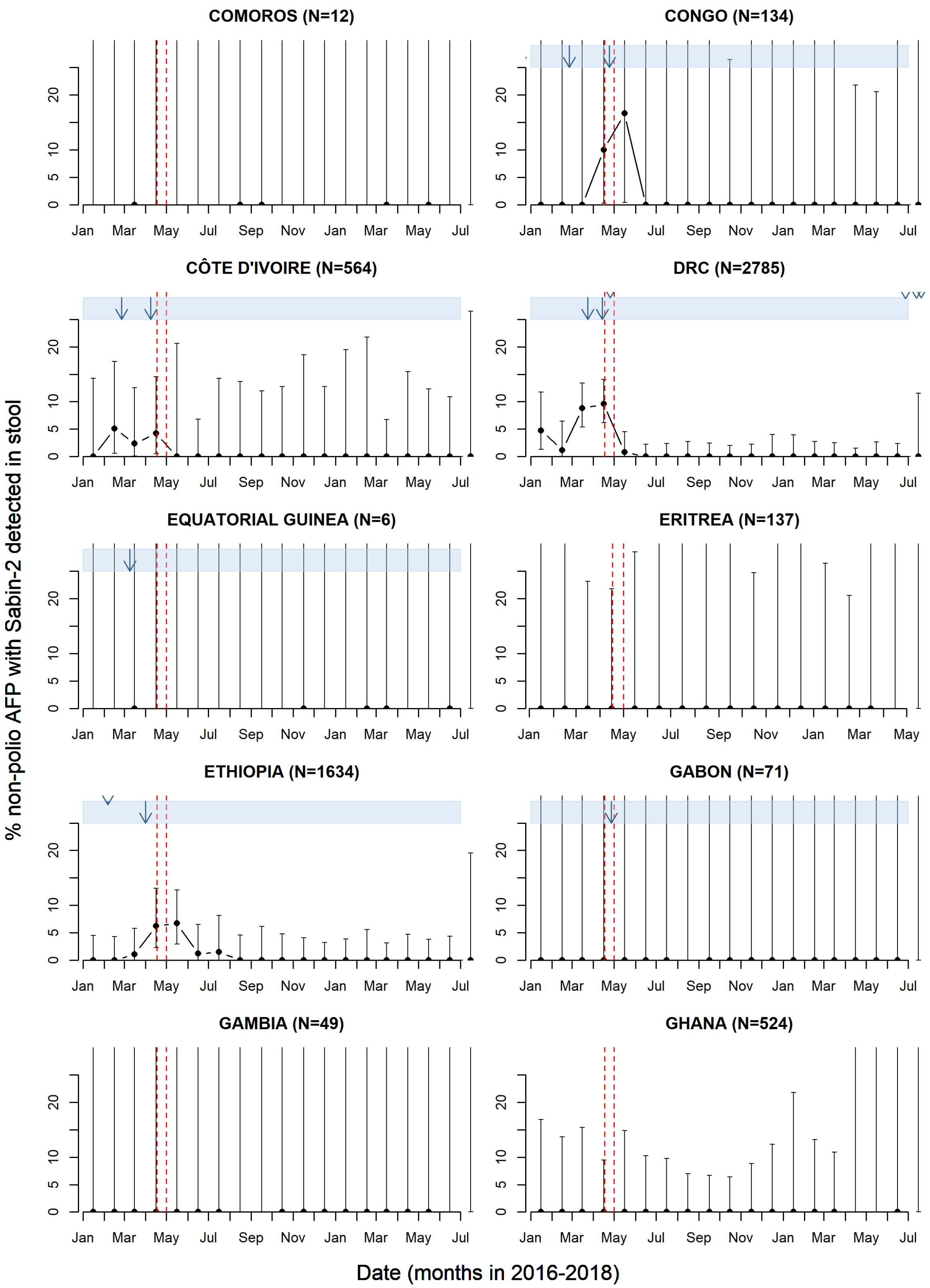
COMOROS (N=12)
CONGO (N=134)
CÔTE D'IVOIRE (N=564)
DRC (N=2785)
EQUATORIAL GUINEA (N=6)
ERITREA (N=137)
ETHIOPIA (N=1634)
GABON (N=71)
GAMBIA (N=49)
GHANA (N=524)
% non-polio AFP with Sabin-2 detected in stool
0
5
10
20
Jan
Mar
May
Jul
Sep
Nov
Date (months in 2016-2018)

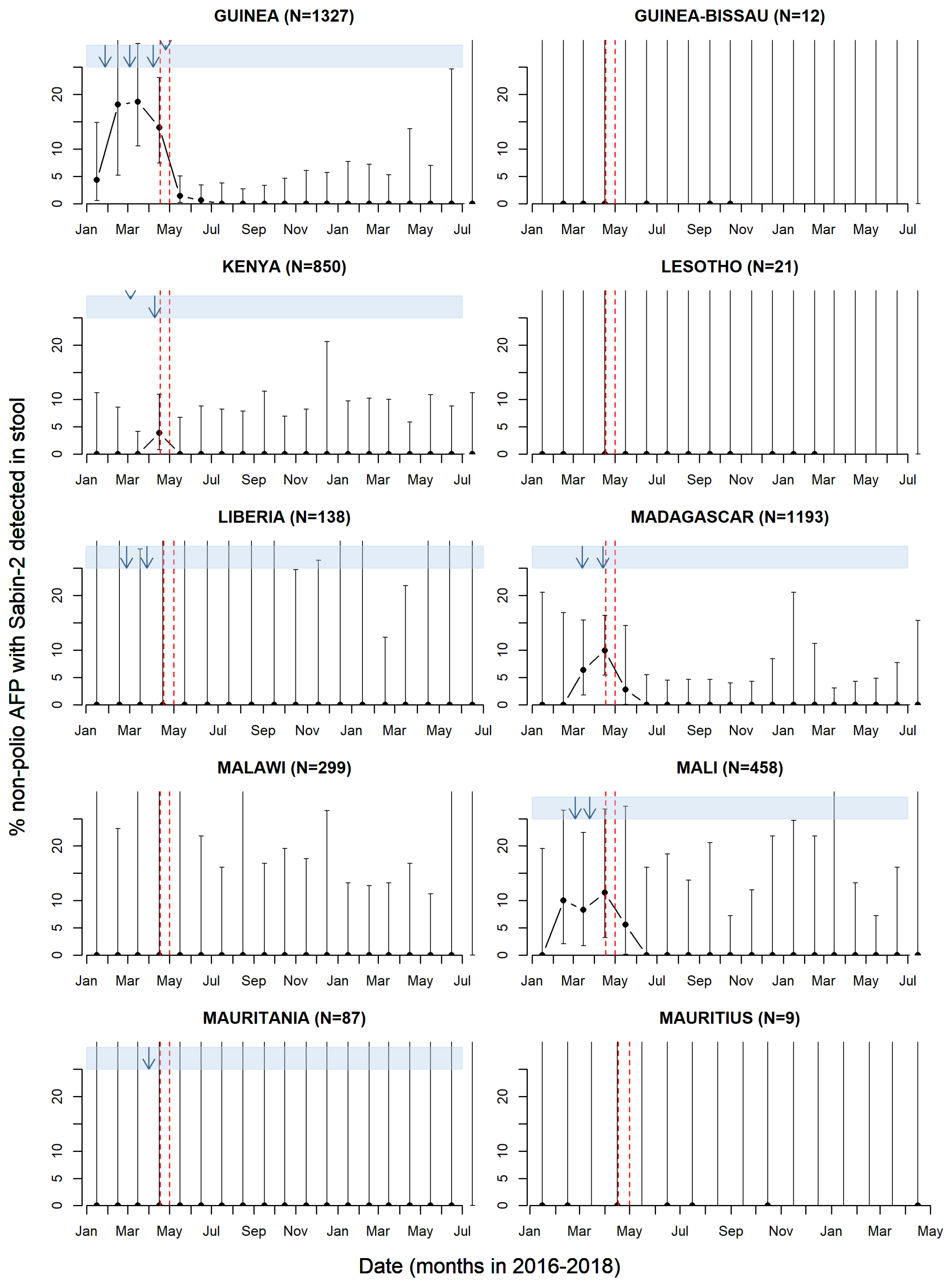

GUINEA (N=1327)
GUINEA-BISSAU (N=12)
KENYA (N=850)
LESOTHO (N=21)
LIBERIA (N=138)
MADAGASCAR (N=1193)
MALAWI (N=299)
MALI (N=458)
MAURITANIA (N=87)
MAURITIUS (N=9)
% non-polio AFP with Sabin-2 detected in stool
0
5
10
20
Jan
Mar
May
Jul
Sep
Nov
Date (months in 2016-2018)

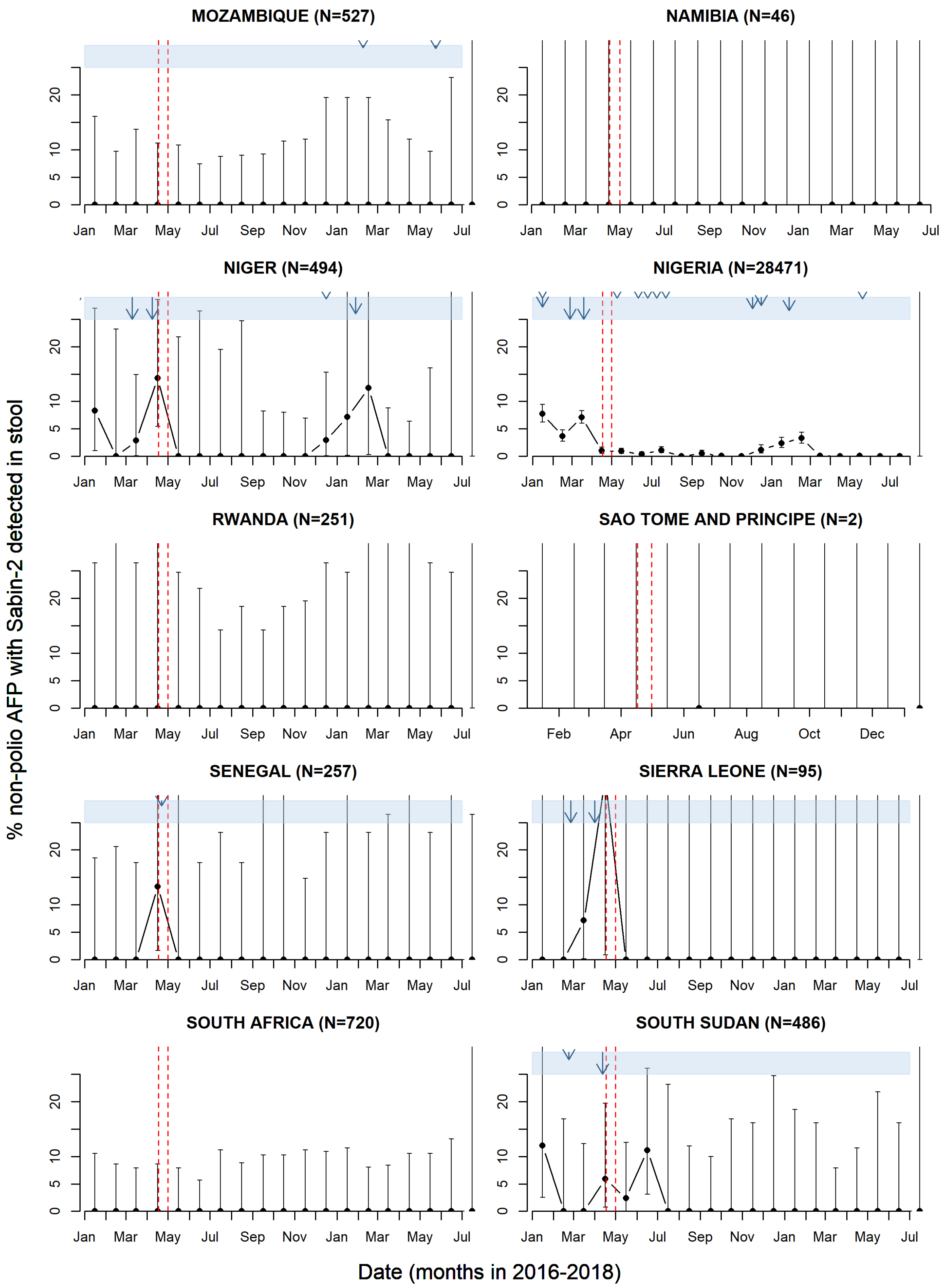
MOZAMBIQUE (N=527)
NAMIBIA (N=46)
NIGER (N=494)
NIGERIA (N=28471)
RWANDA (N=251)
SAO TOME AND PRINCIPE (N=2)
SENEGAL (N=257)
SIERRA LEONE (N=95)
SOUTH AFRICA (N=720)
SOUTH SUDAN (N=486)
% non-polio AFP with Sabin-2 detected in stool
Date (months in 2016-2018)

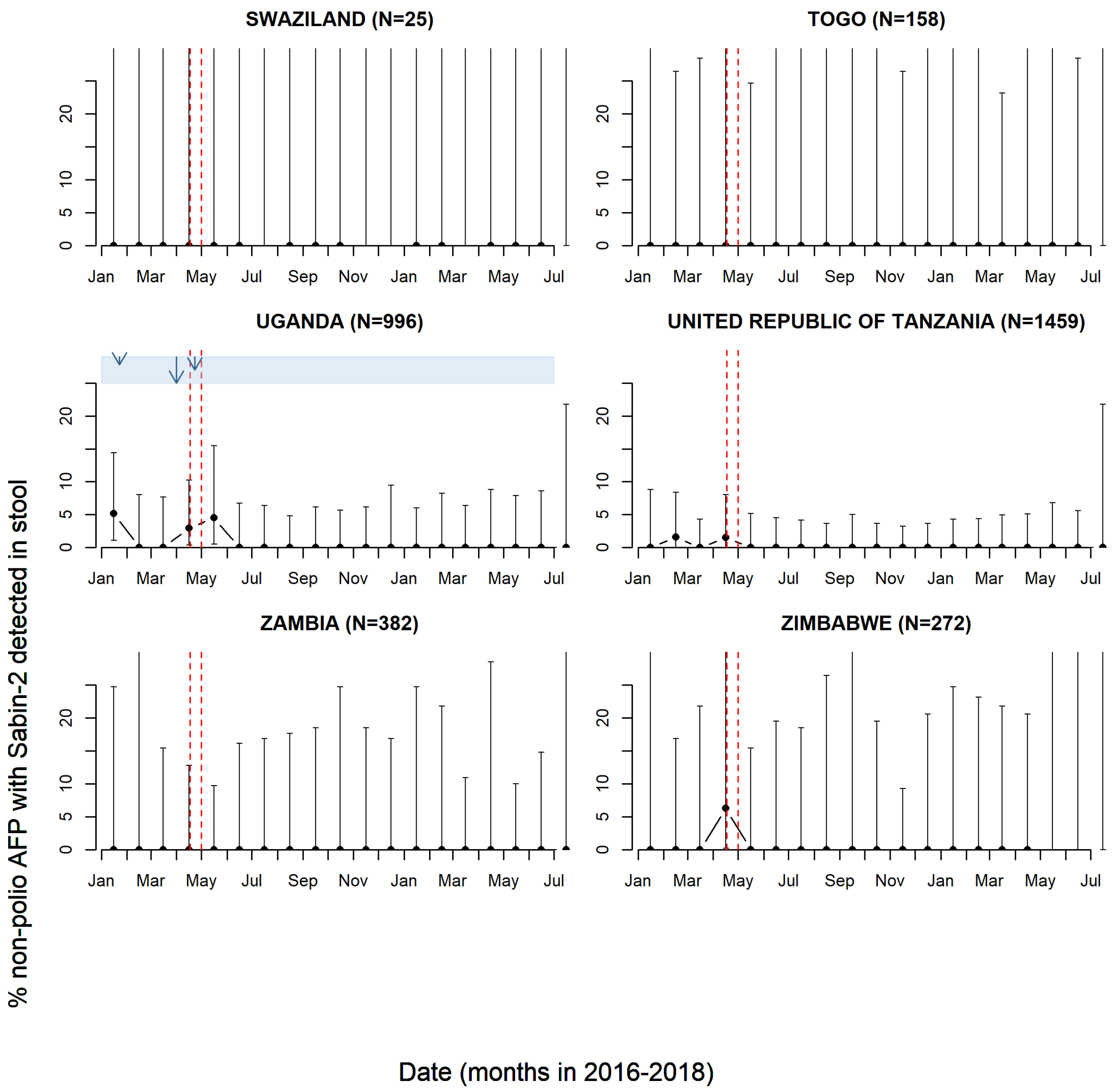

Figure S3 Percentage of monthly non-polio AFP with Sabin-2 detected in stool (line graphs) by country in the African region. Error bars indicate 95% binomial confidence intervals. The red dashed lines indicate the timing of OPV2 withdrawal and the blue arrows indicate whether a campaign occurred with tOPV (prior to OPV2 withdrawal) or mOPV2 post OPV2 withdrawal. The length or the arrow is proportional to the fraction of the population targeted at each campaign and the height of the blue box indicates 100% of the population targeted

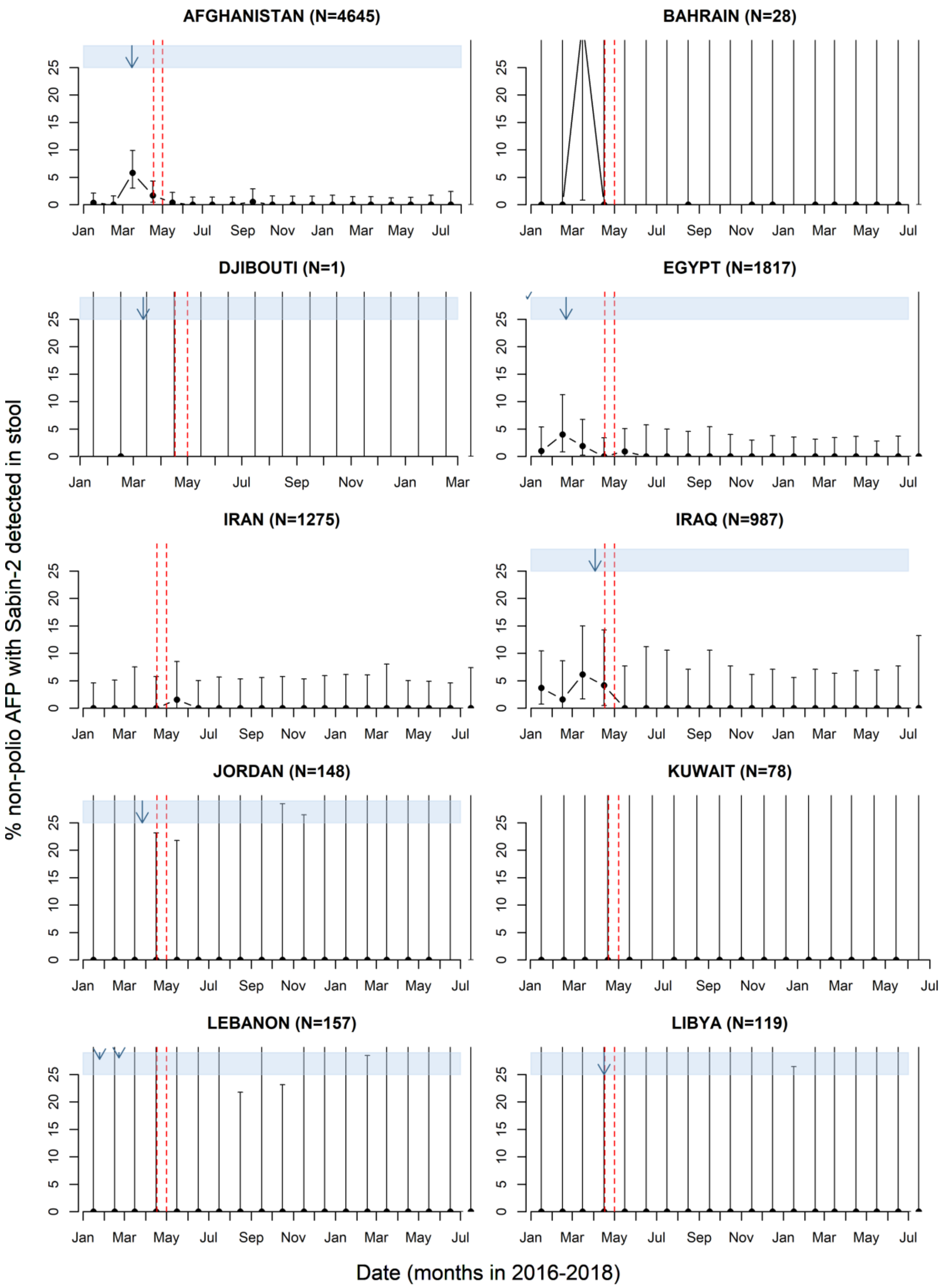
AFGHANISTAN (N=4645)
BAHRAIN (N=28)
DJIBOUTI (N=1)
EGYPT (N=1817)
IRAN (N=1275)
IRAQ (N=987)
JORDAN (N=148)
KUWAIT (N=78)
LEBANON (N=157)
LIBYA (N=119)
% non-polio AFP with Sabin-2 detected in stool
Date (months in 2016-2018)

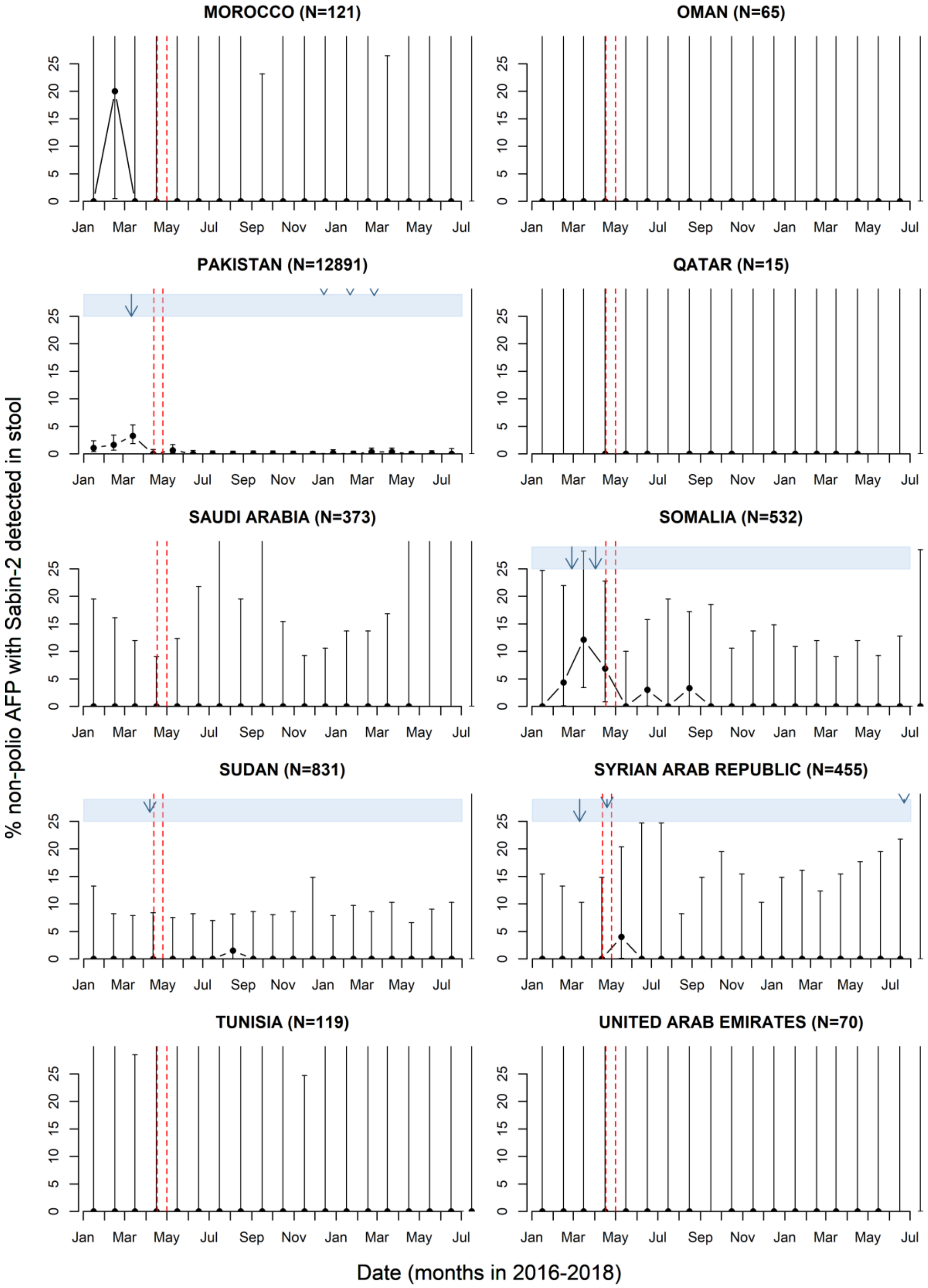
MOROCCO (N=121)
OMAN (N=65)
PAKISTAN (N=12891)
QATAR (N=15)
SAUDI ARABIA (N=373)
SOMALIA (N=532)
SUDAN (N=831)
SYRIAN ARAB REPUBLIC (N=455)
TUNISIA (N=119)
UNITED ARAB EMIRATES (N=70)
% non-polio AFP with Sabin-2 detected in stool
Date (months in 2016-2018)
Jan Mar May Jul Sep Nov Jan Mar May Jul
0 5 10 15 20 25

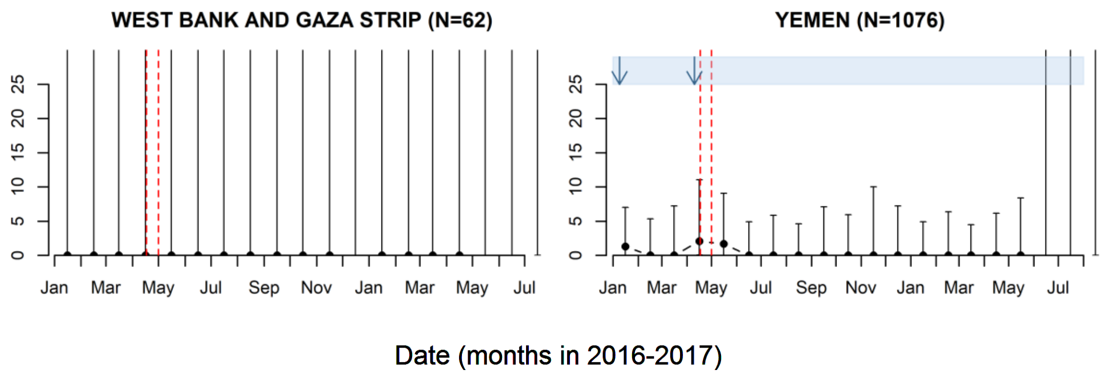

Figure S4 Percentage of monthly non-polio AFP with Sabin-2 detected in stool (line graphs) by country in the Eastern Mediterranean region. Error bars indicate 95% binomial confidence intervals. The red dashed lines indicate the timing of OPV2 withdrawal and the blue arrows indicate whether a campaign occurred with tOPV (prior to OPV2 withdrawal) or mOPV2 post OPV2 withdrawal. The length or the arrow is proportional to the fraction of the population targeted at each campaign and the height of the blue box indicates 100% of the population targeted

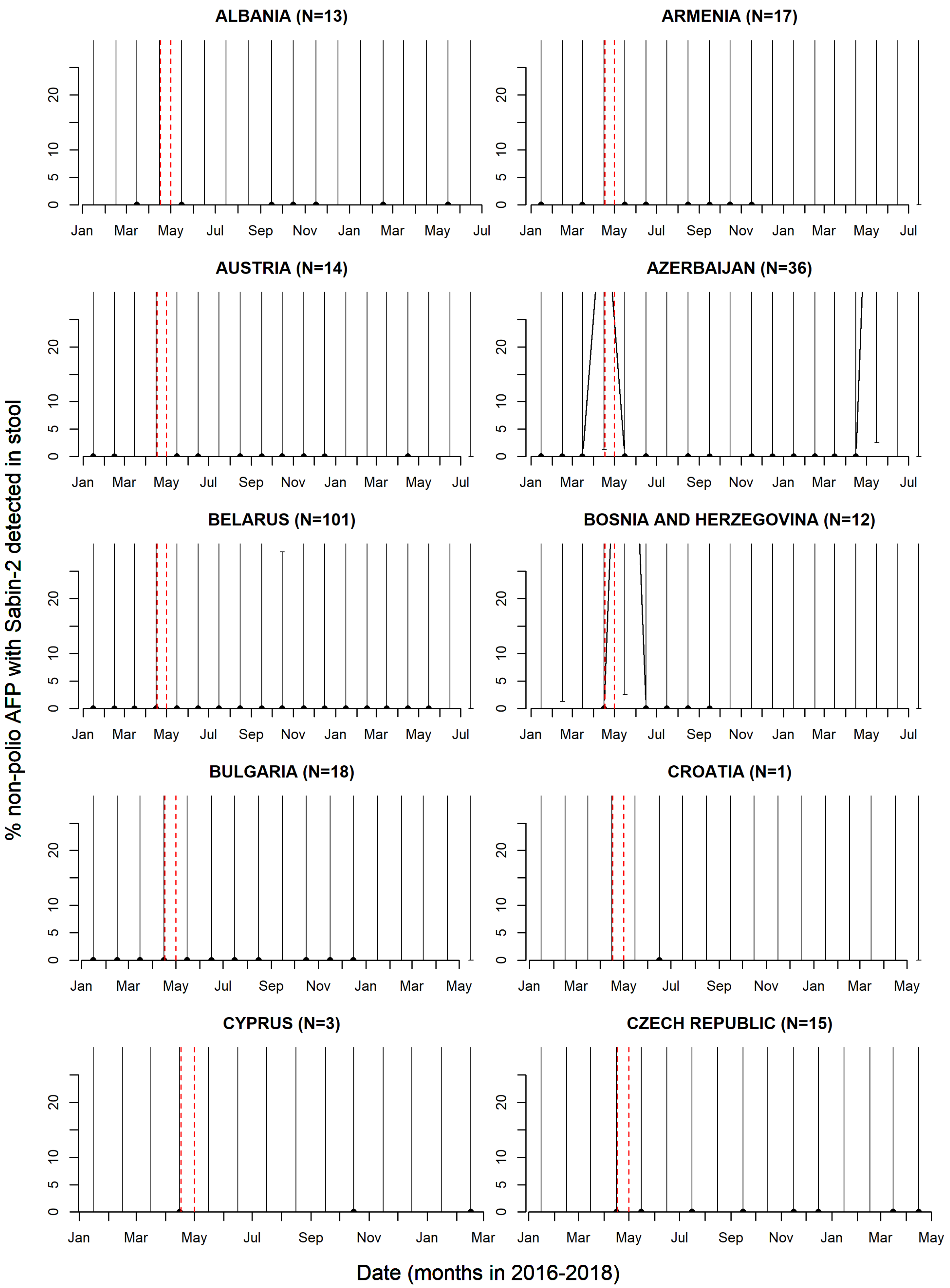
ALBANIA (N=13)
ARMENIA (N=17)
AUSTRIA (N=14)
AZERBAIJAN (N=36)
BELARUS (N=101)
BOSNIA AND HERZEGOVINA (N=12)
BULGARIA (N=18)
CROATIA (N=1)
CYPRUS (N=3)
CZECH REPUBLIC (N=15)
% non-polio AFP with Sabin-2 detected in stool
Date (months in 2016-2018)

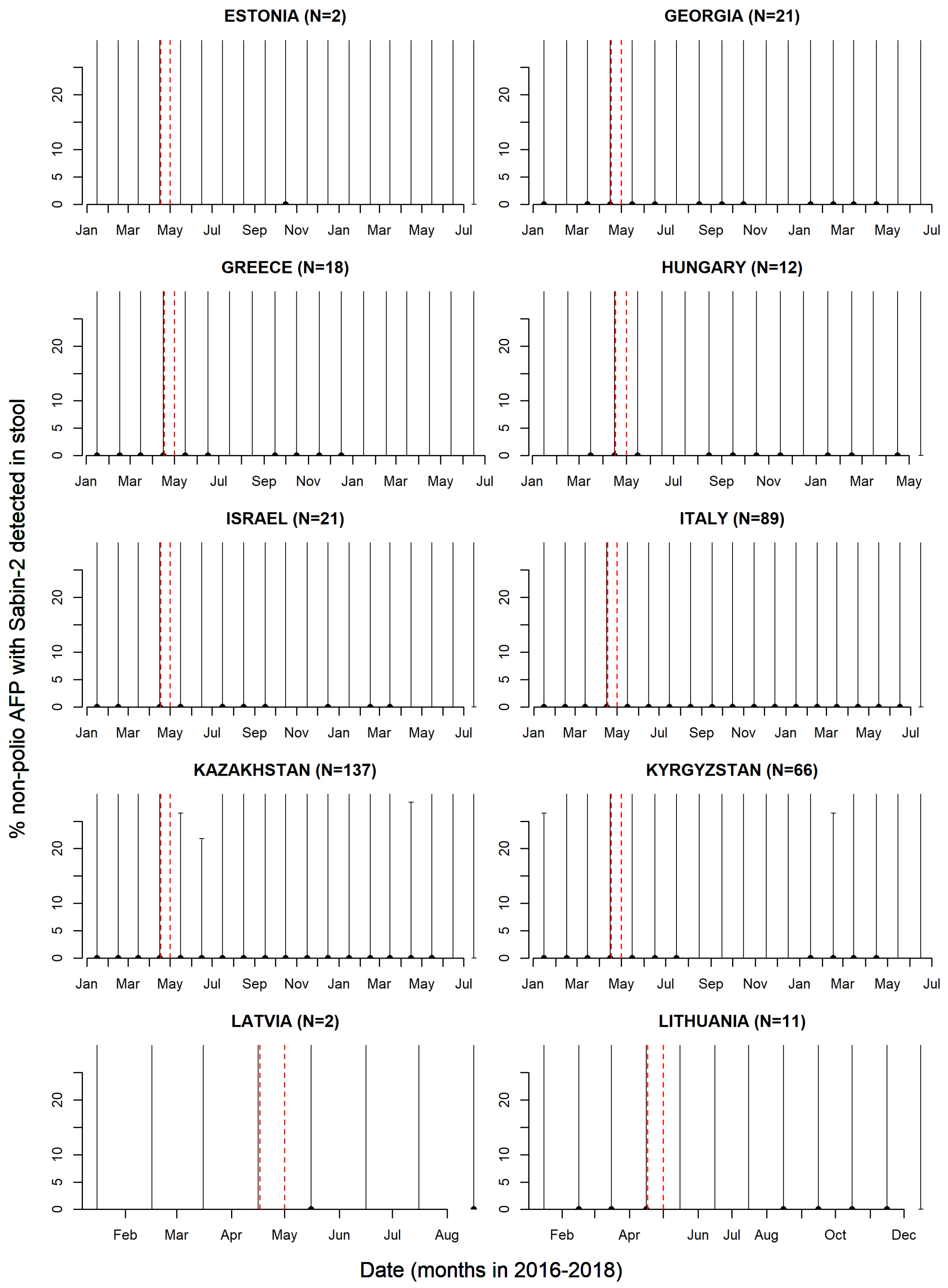
ESTONIA (N=2)
GEORGIA (N=21)
GREECE (N=18)
HUNGARY (N=12)
ISRAEL (N=21)
ITALY (N=89)
KAZAKHSTAN (N=137)
KYRGYZSTAN (N=66)
LATVIA (N=2)
LITHUANIA (N=11)
% non-polio AFP with Sabin-2 detected in stool
Date (months in 2016-2018)

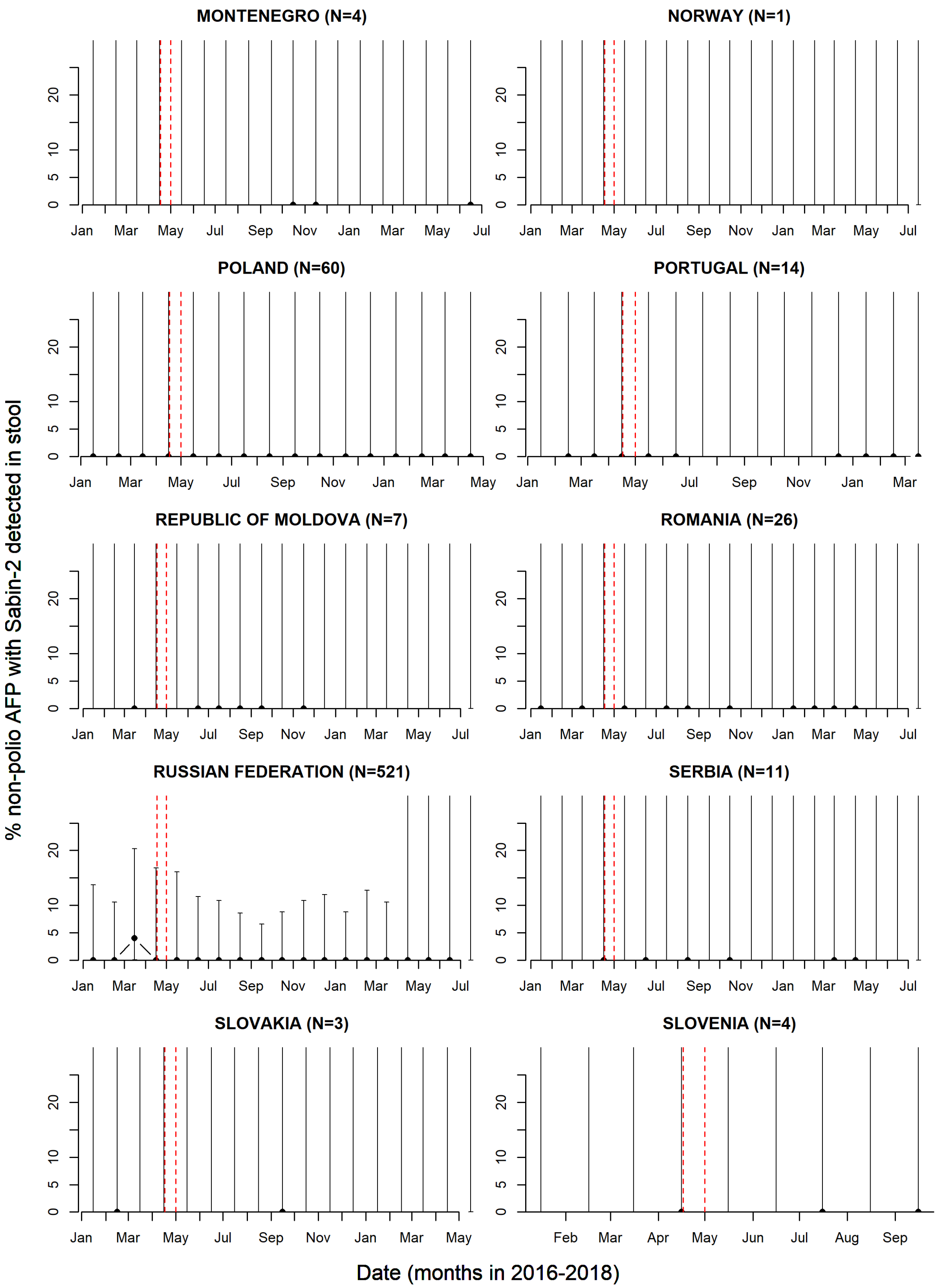

MONTENEGRO (N=4)
NORWAY (N=1)
POLAND (N=60)
PORTUGAL (N=14)
REPUBLIC OF MOLDOVA (N=7)
ROMANIA (N=26)
RUSSIAN FEDERATION (N=521)
SERBIA (N=11)
SLOVAKIA (N=3)
SLOVENIA (N=4)
% non-polio AFP with Sabin-2 detected in stool
Date (months in 2016-2018)

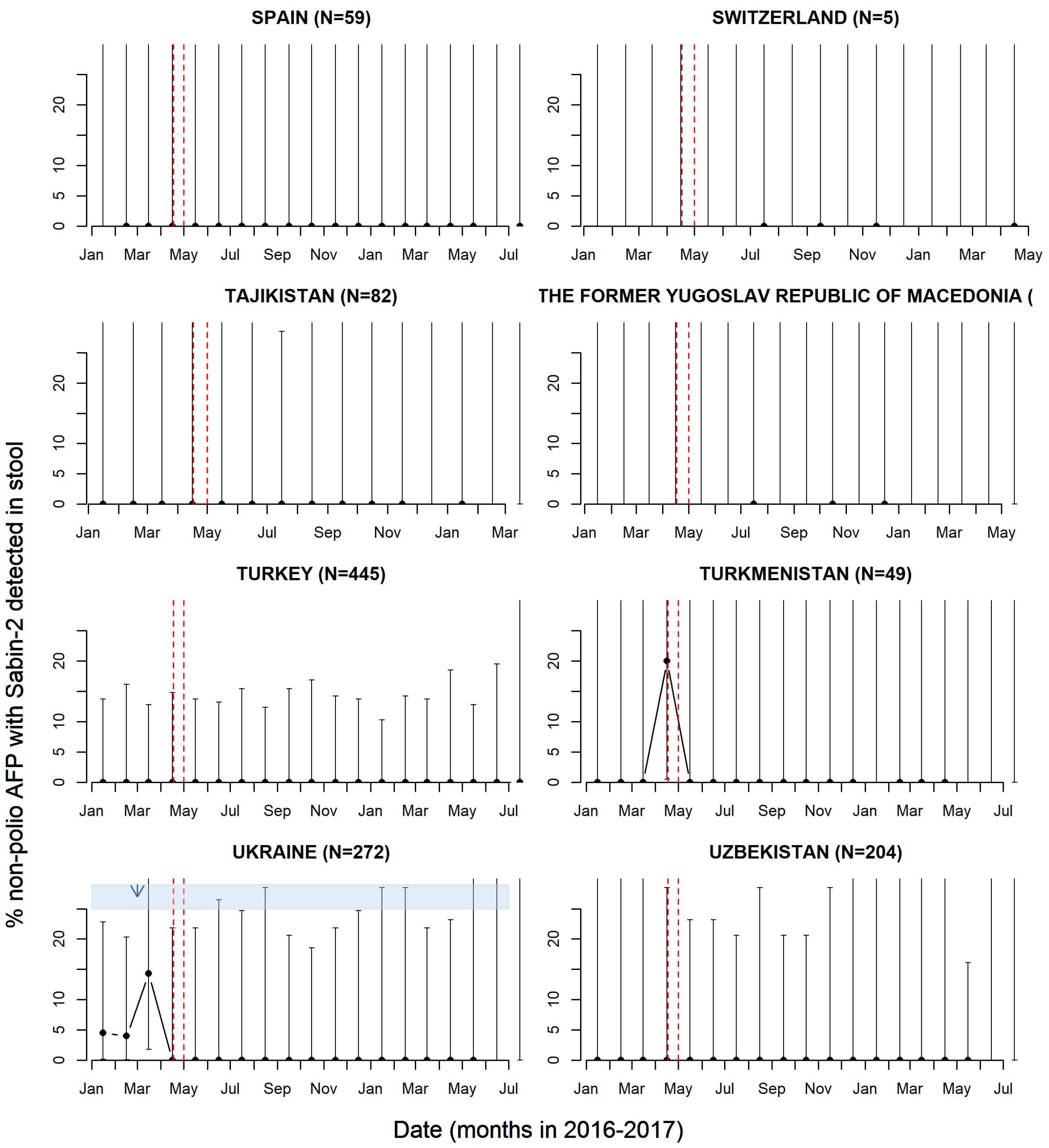

Figure S5 Percentage of monthly non-polio AFP with Sabin-2 detected in stool (line graphs) by country in the European region. Error bars indicate 95% binomial confidence intervals. The red dashed lines indicate the timing of OPV2 withdrawal and the blue arrows indicate whether a campaign occurred with tOPV (prior to OPV2 withdrawal) or mOPV2 post OPV2 withdrawal. The length or the arrow is proportional to the fraction of the population targeted at each campaign and the height of the blue box indicates 100% of the population targeted

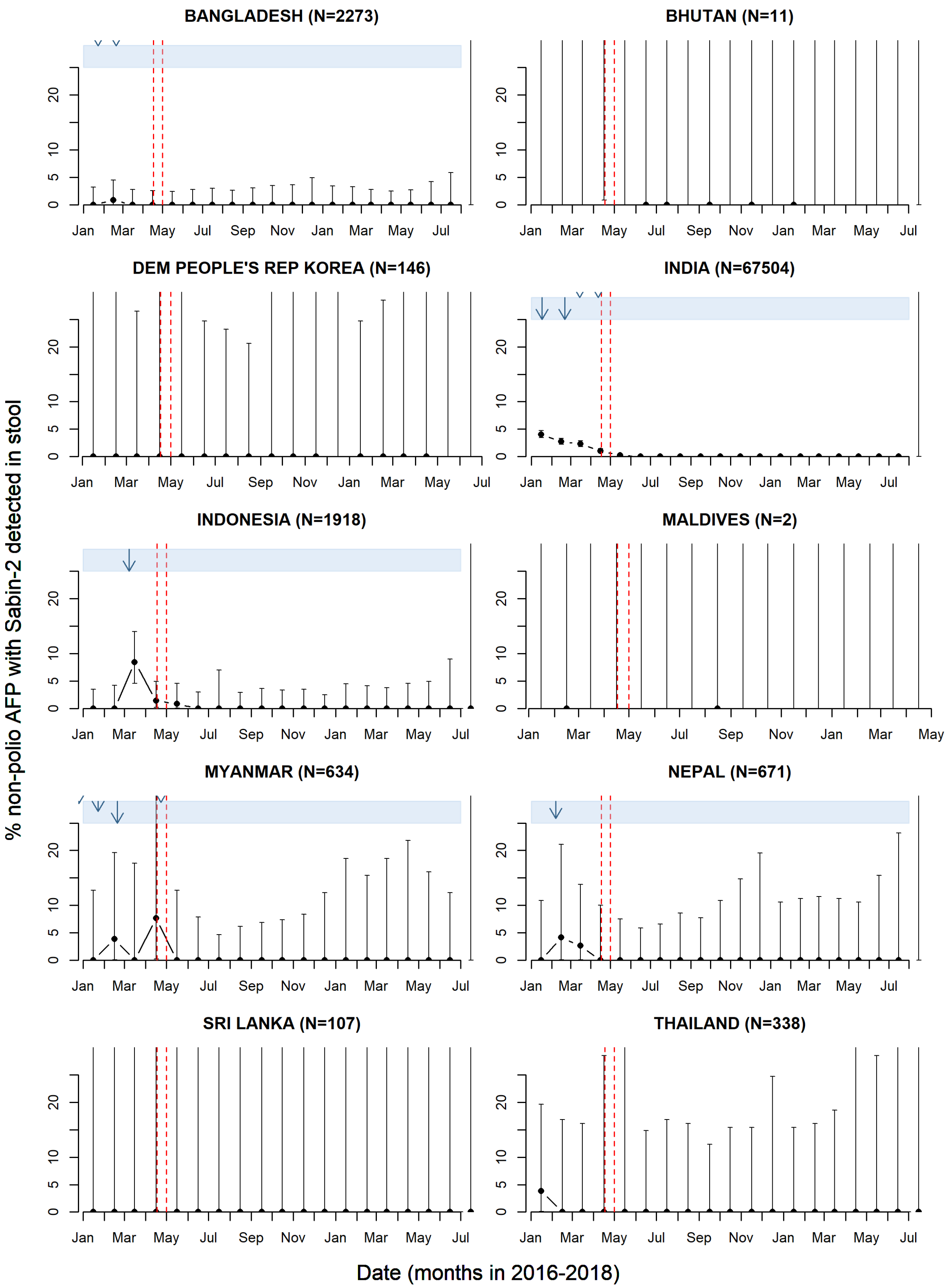

BANGLADESH (N=2273)
BHUTAN (N=11)
DEM PEOPLE'S REP KOREA (N=146)
INDIA (N=67504)
INDONESIA (N=1918)
MALDIVES (N=2)
MYANMAR (N=634)
NEPAL (N=671)
SRI LANKA (N=107)
THAILAND (N=338)
% non-polio AFP with Sabin-2 detected in stool
Date (months in 2016-2018)

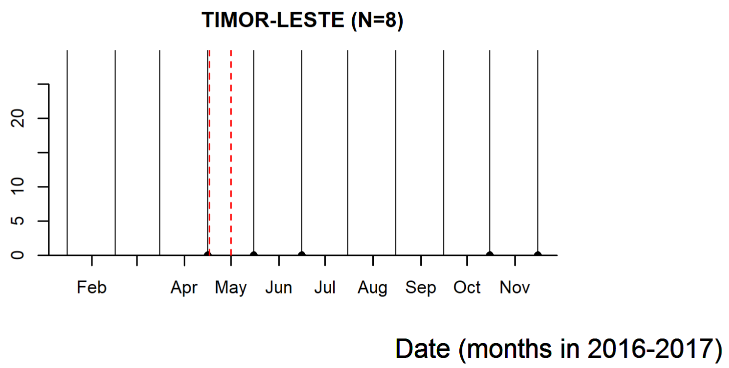

Figure S6 Percentage of monthly non-polio AFP with Sabin-2 detected in stool (line graphs) by country in the South-East Asia region. Error bars indicate 95% binomial confidence intervals. The red dashed lines indicate the timing of OPV2 withdrawal and the blue arrows indicate whether a campaign occurred with tOPV (prior to OPV2 withdrawal) or mOPV2 post OPV2 withdrawal. The length or the arrow is proportional to the fraction of the population targeted at each campaign and the height of the blue box indicates 100% of the population targeted

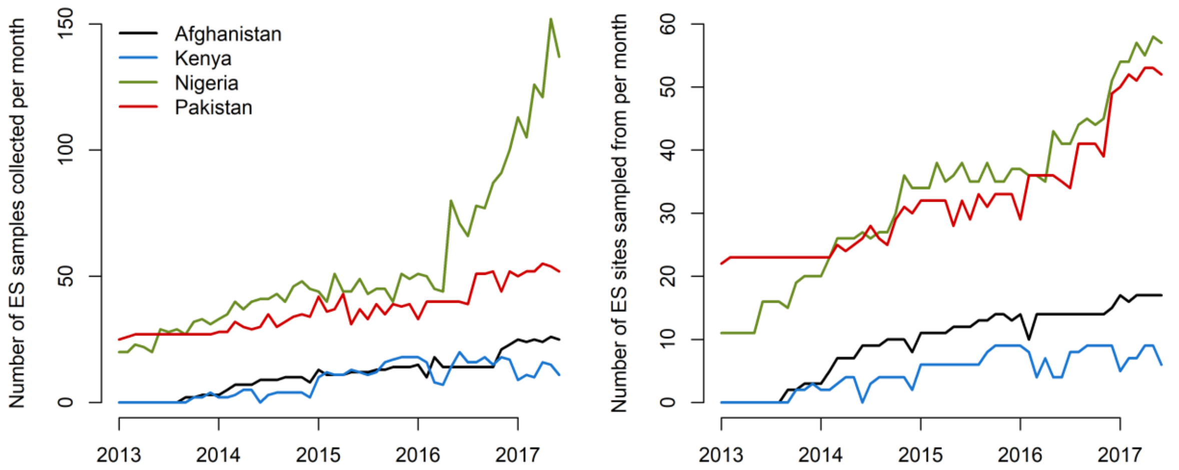

Figure S7 Number of ES samples collected per month and the number of sites sampled from by country as of 08 August 2017

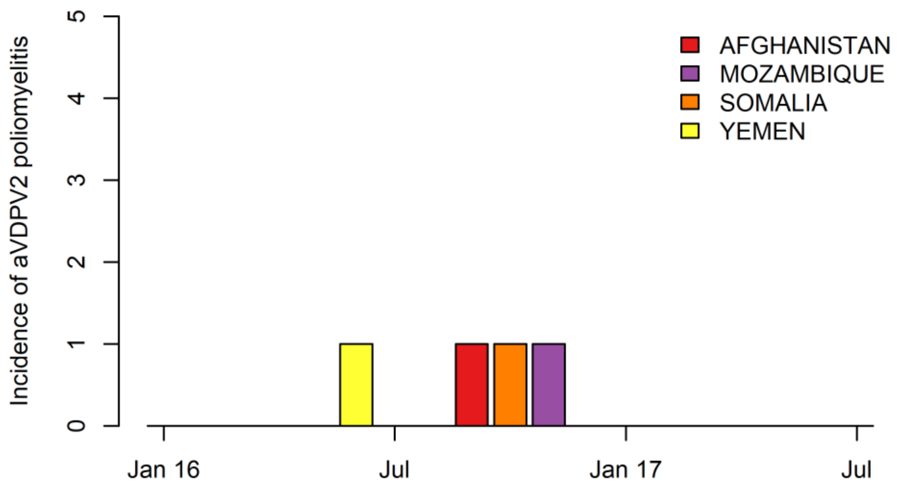

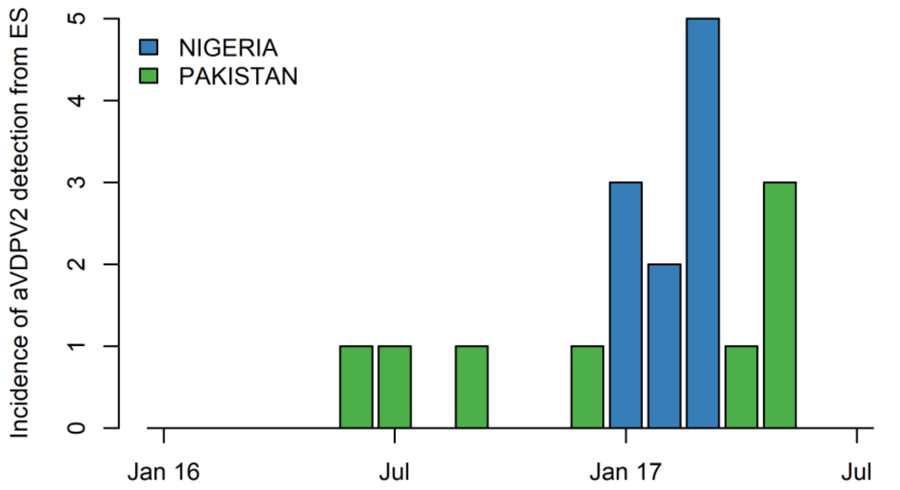

Figure S8 Monthly incidence of ambiguous type-2 vaccine-derived poliovirus.

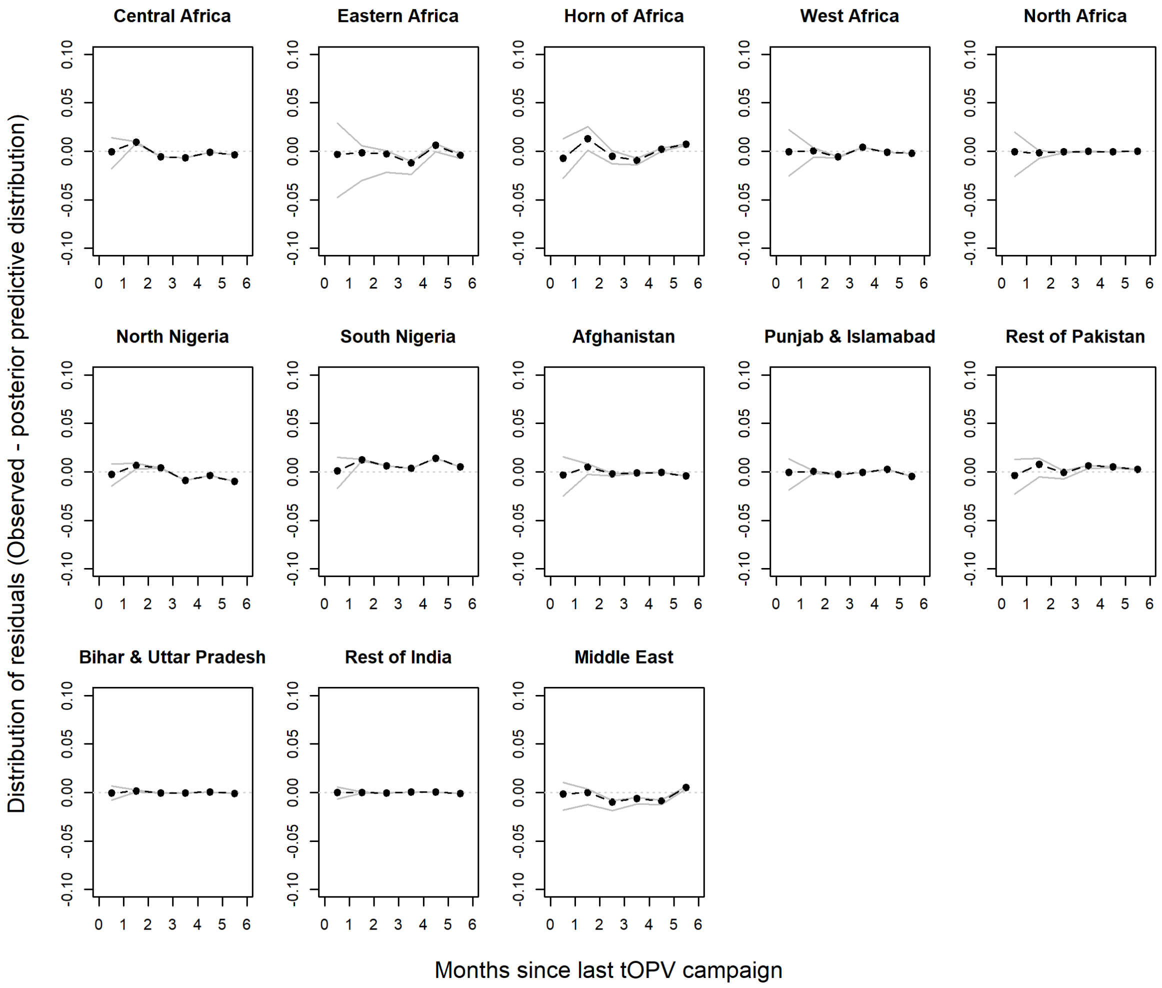

Figure S9 Distribution of residuals (Observed Sabin-2 detection from non-polio AFP cases – posterior predictive distribution), aggregated by month since the last tOPV campaign. The black points denote the mean and grey lines denote the 95% credible interval.

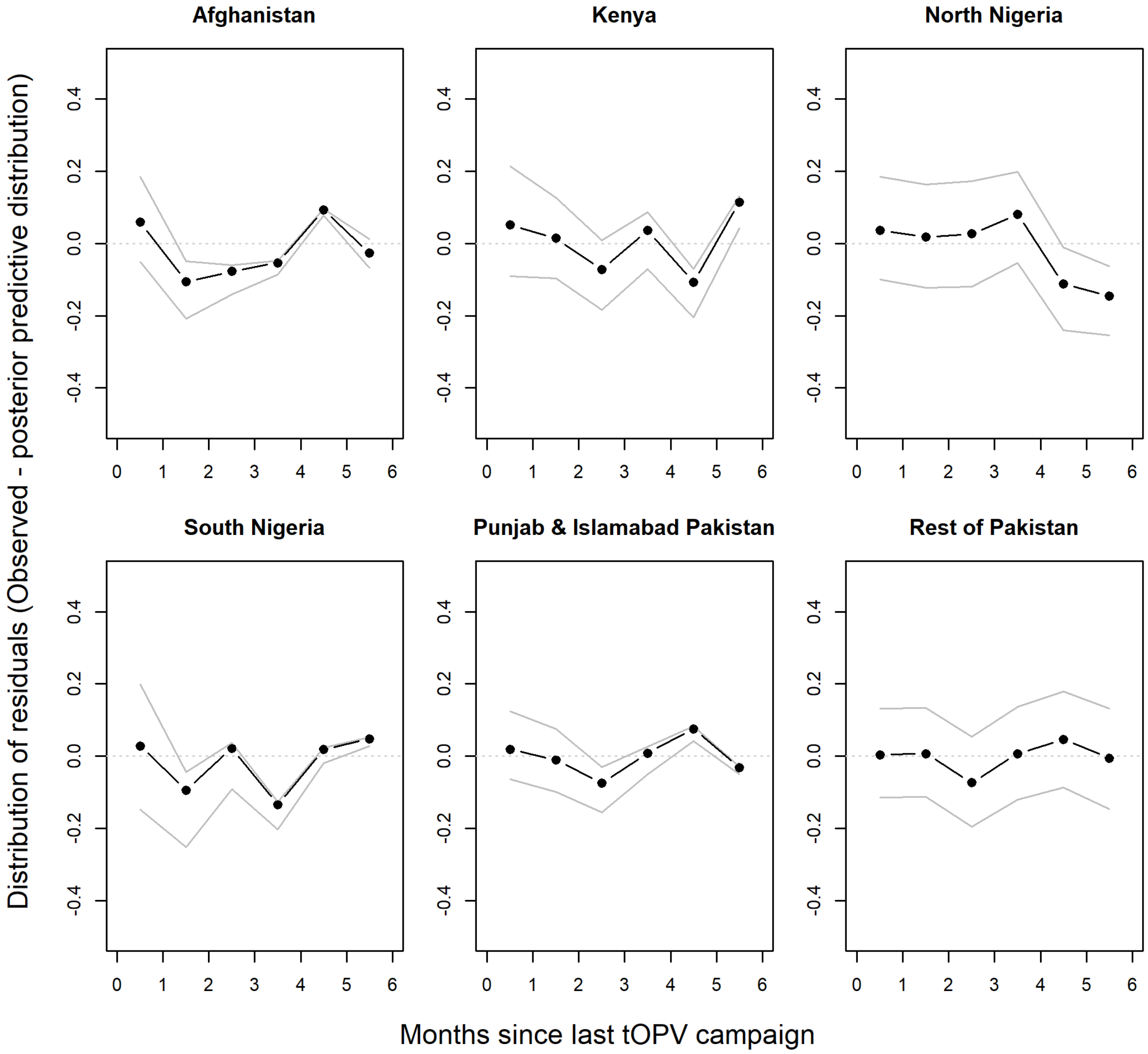

Figure S10 Distribution of residuals (Observed Sabin-2 detection from sewage samples – posterior predictive distribution), aggregated by month since the last tOPV campaign. The black points denote the mean and grey lines denote the 95% credible interval.

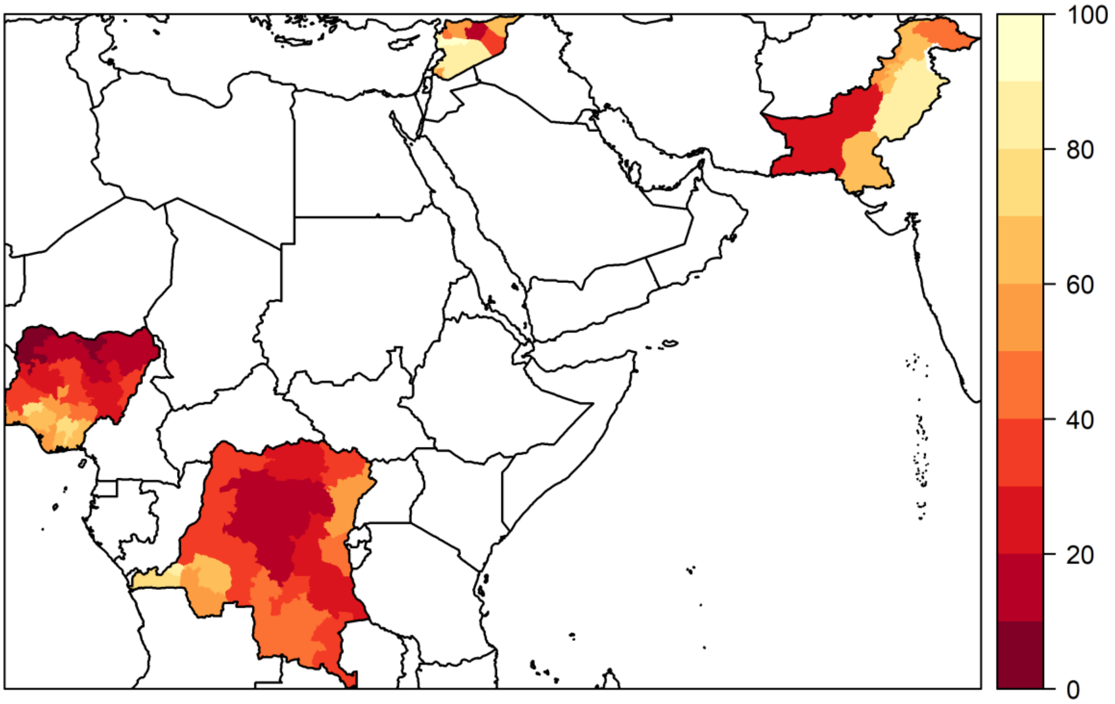

Figure S11 Estimates of routine immunisation coverage (%) at the province level for countries that reported cVDPV2 poliomyelitis cases after OPV2 withdrawal. Estimates for Pakistan and Syria were obtained from reported number of OPV doses of OPV received through routine immunisation by non-polio AFP data aged 12-23 months (reporting paralysis in the first half of 2017). Estimates for DRC and Nigeria were obtained from the most recent Demographic Health Survey (2013) based on reported DTP3 coverage.

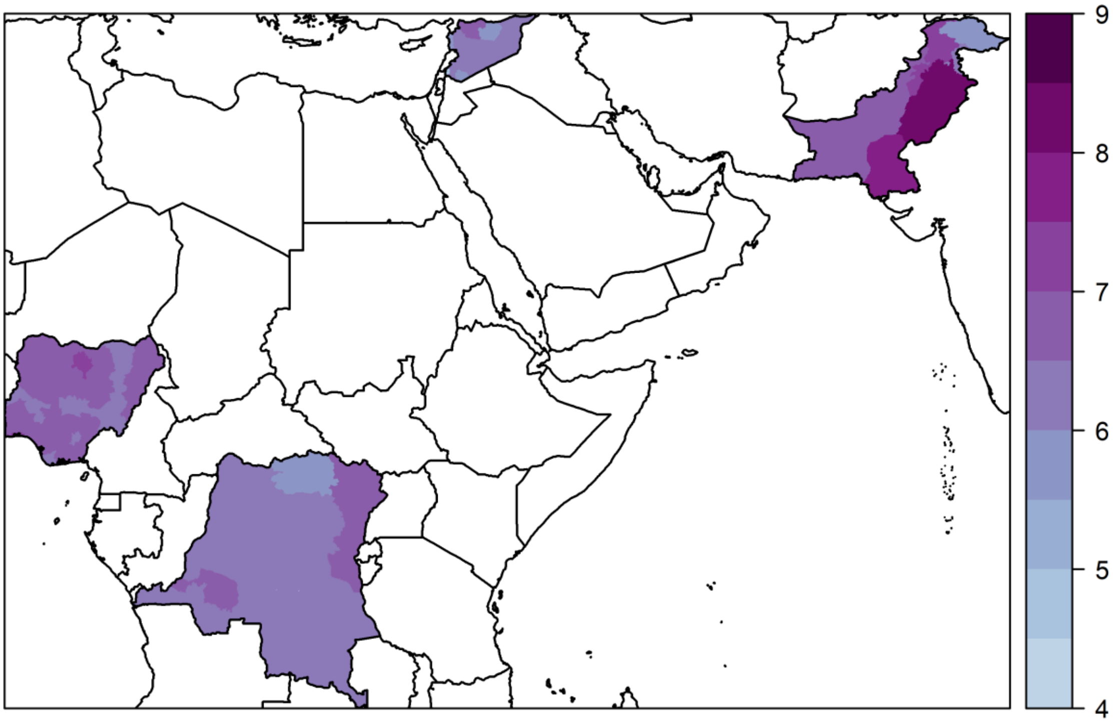

Figure S12 Estimates of log10 total population size at the province level for countries that reported cVDPV2 poliomyelitis cases after OPV2 withdrawal. Estimates were obtained from the WorldPop project (2015).

References

1. Levitt A, Diop OM, Tangermann RH, et al. Surveillance systems to track progress toward global polio eradication - worldwide, 2012-2013. MMWR Morb Mortal Wkly Rep 2014;63:356-61.
2. Polio Laboratory manual. 2004. (Accessed September, 2017, at http://polioeradication.org/wp-content/uploads/2017/05/Polio_Lab_Manual04.pdf.)
3. S1 Supplement to the WHO polio laboratory manual (Accessed September, 2017, at http://polioeradication.org/wp-content/uploads/2017/05/NewAlgorithmForPoliovirusIsolationSupplement1.pdf.)
4. Grassly NC, Wenger J, Durrani S, et al. Protective efficacy of a monovalent oral type 1 poliovirus vaccine: a case-control study. Lancet 2007;369:1356-62.
5. Asghar H, Diop OM, Weldegebriel G, et al. Environmental surveillance for polioviruses in the Global Polio Eradication Initiative. The Journal of infectious diseases 2014;210 Suppl 1:S294-303.
6. Polio Environmental Surveillance Expansion Plan. 2015. at http://polioeradication.org/wp-content/uploads/2016/07/GPLN_ExpansionPlanES.pdf.)
7. Johnson Muluh T, Hamisu AW, Craig K, et al. Contribution of Environmental Surveillance Toward Interruption of Poliovirus Transmission in Nigeria, 2012-2015. The Journal of infectious diseases 2016;213 Suppl 3:S131-5.
8. Guidelines on environmental surveillance for detection of polioviruses. 2015. (Accessed 04 October, 2016, at http://polioeradication.org/wp-content/uploads/2016/07/GPLN_GuidelinesES_April2015.pdf.)
9. Fagnant CS, Beck NK, Yang MF, Barnes KS, Boyle DS, Meschke JS. Development of a novel bag-mediated filtration system for environmental recovery of poliovirus. J Water Health 2014;12:747-54.
10. Pons-Salort M, Molodecky NA, O'Reilly KM, et al. Population immunity against serotype-2 poliomyelitis leading up to the global withdrawal of the oral poliovirus vaccine: Spatio-temporal modelling of surveillance data. PLoS medicine 2016;13:e1002140.
11. Gelman A, Goegebeur Y, Tuerlinckx F, Van Mechelen I. Diagnostic checks for discrete data regression models using posterior predictive simulations. J Roy Stat Soc C-App 2000;49:247-68.
12. Worldpop. 2017. (Accessed 30 October, 2017, at www.worldpop.org.uk.)
13. Carnell R. lhs: Latin Hypercube Samples. R package version 0.14. 2016.
14. Pons-Salort M, Burns CC, Lyons H, et al. Preventing Vaccine-Derived Poliovirus Emergence during the Polio Endgame. PLoS Pathog 2016;12:e1005728.
15. Molodecky NA, Blake IM, O'Reilly KM, et al. Risk factors and short-term projections for serotype-1 poliomyelitis incidence in Pakistan: A spatiotemporal analysis. PLoS medicine 2017;14:e1002323.
16. Modeled Surfaces. ICF International. (Accessed 26 October, 2017, at http://spatialdata.dhsprogram.com.)
17. Hunziker P. velox: Fast Raster Manipulation and Extraction. R package version 0.1.0. 2016.
18. Linard C, Gilbert M, Snow RW, Noor AM, Tatem AJ. Population distribution, settlement patterns and accessibility across Africa in 2010. PloS one 2012;7:e31743.
19. Watanabe S. Asymptomatic equivalence of Bayes cross validation and widely applicable information criterion in sinhular learning theory. Jounral of Machine Learning Research 2010;11:3571 - 94.
20. Rue H, Martino S, Lindgren F, Simpson D, Riebler A, T. KE. INLA: Functions which allow to perform full Bayesian analysis of latent Gaussian models using Integrated Nested Laplace Approximation. R package version 0.0-1403203700. 2014.
21. Jorba J, Diop OM, Iber J, et al. Update on Vaccine-Derived Polioviruses - Worldwide, January 2016-June 2017. MMWR Morb Mortal Wkly Rep 2017;66:1185-91.
